# Oxidized SOD1 accelerates cellular senescence in neural stem cells

**DOI:** 10.1186/s13287-024-03669-5

**Published:** 2024-02-27

**Authors:** Teng Guan, Ying Guo, Ting Zhou, Qiang Yu, Jingyi Sun, Baoliang Sun, Guohui Zhang, Jiming Kong

**Affiliations:** 1 Department of Human Anatomy and Cell Science, University of Manitoba, Winnipeg, MB Canada; 2 Department of Forensic Medicine, Hebei North University, Zhangjiakou, Hebei China; 3 Department of Pharmacy, The Second Affiliated Hospital of Chongqing Medical University, Chongqing, China; 4 Shandong First Medical University & Shandong Academy of Medical Sciences, Jinan, 250117 Shandong China

## Abstract

**Background:**

Neural stem cells (NSCs), especially human NSCs, undergo cellular senescence characterized by an irreversible proliferation arrest and loss of stemness after prolonged culture. While compelling correlative data have been generated to support the oxidative stress theory as one of the primary determinants of cellular senescence of NSCs, a direct cause-and-effect relationship between the accumulation of oxidation-mediated damage and cellular senescence of NSCs has yet to be firmly established. Human SOD1 (hSOD1) is susceptible to oxidation. Once oxidized, it undergoes aberrant misfolding and gains toxic properties associated with age-related neurodegenerative disorders. The present study aims to examine the role of oxidized hSOD1 in the senescence of NSCs.

**Methods:**

NSCs prepared from transgenic mice expressing the wild-type *hSOD1* gene were maintained in culture through repeated passages. Extracellular vesicles (EVs) were isolated from culture media at each passage. To selectively knock down oxidized SOD1 in NSCs and EVs, we used a peptide-directed chaperone-mediated protein degradation system named CT4 that we developed recently.

**Results:**

In NSCs expressing the *hSOD1* from passage 5, we detected a significant increase of oxidized hSOD1 and an increased expression of biomarkers of cellular senescence, including upregulation of P53 and SA-β-Gal and cytoplasmic translocation of HMGB1. The removal of oxidized SOD1 remarkably increased the proliferation and stemness of the NSCs. Meanwhile, EVs derived from senescent NSCs carrying the wild-type *hSOD1* contained high levels of oxidized hSOD1, which could accelerate the senescence of young NSCs and induce the death of cultured neurons. The removal of oxidized hSOD1 from the EVs abolished their senescence-inducing activity. Blocking oxidized SOD1 on EVs with the SOD1 binding domain of the CT4 peptide mitigated its toxicity to neurons.

**Conclusion:**

Oxidized hSOD1 is a causal factor in the cellular senescence of NSCs. The removal of oxidized hSOD1 is a strategy to rejuvenate NSCs and to improve the quality of EVs derived from senescent cells.

**Supplementary Information:**

The online version contains supplementary material available at 10.1186/s13287-024-03669-5.

## Introduction

Adult neural stem cells (NSCs) are present in the subgranular zone of the dentate gyrus, the subventricular zone of the lateral ventricles [1] and the ependymal layer in the spinal cord [2]. These cells are typically maintained in quiescence but can be activated to enter the cell cycle [3]. Emerging observations suggest that proteostasis plays a role in maintaining NSC quiescence [4]. Clearance of protein aggregates activates NSCs [5]. Upon aging, NSCs undergo proliferative declines and cellular senescence due to the accumulation of protein aggregates [6]. To counteract these effects, stimulation of lysosomal activity to enhance the degradation of protein aggregates rejuvenates aged NSCs [7].

Superoxide dismutase 1 (SOD1) is an antioxidant responsible for neutralizing supercharged oxygen radicals in the cell. It exists predominantly in the cytoplasm, although a small portion of SOD1 monomers is present in the mitochondrial inner membrane space to scavenge superoxide ions generated from the mitochondrial respiration chain. Deficiency in SOD1 leads to oxidative stress in the cell [8] and accelerates age-associated behavioural abnormalities [9]. Interestingly, SOD1 is susceptible to oxidative modifications and can even be oxidized by hydrogen peroxide [10], its catalytic reaction product. Mutations in the human *SOD1* gene increase its susceptibility to oxidation and are a cause of familial amyotrophic lateral sclerosis (ALS), an age-related neurodegenerative disease [11–13]. The cysteine 111 is particularly susceptible to oxidation [10, 12]. Oxidatively modified SOD1 adopts a misfolded conformation that exposes its hydrophobic sequences to interact covalently with the exposed hydrophobic patches of other misfolded hSOD1. Such oligomers offer covalent attachment to other native proteins and form high molecular weight aggregates [10, 12]. As such, oxidation of SOD1 triggers aberrant misfolding and protein aggregation. Meanwhile, oxidized hSOD1 tends to localize to mitochondrial membranes and causes mitochondrial dysfunction [14, 15]. Oxidation of SOD1 has, therefore, been suggested to play a role in aging and age-related diseases [16, 17].

Here, we report that oxidized hSOD1 accumulates in NSCs expressing hSOD1 after repeated passages in culture. With the accumulation of oxidized hSOD1, NSCs undergo early entrance into replicative senescence. Based on chaperone-mediated autophagy (CMA), which targets cytosolic proteins bearing a CMA targeting motif (CTM) for lysosomal degradation, we have developed a peptide-directed CMA-mediated protein degradation system named CT4 to degrade misfolded SOD1 selectively. As reported recently[18], the CT4 system could robustly knock down misfolded SOD1 in vitro and in vivo. Since all oxidized SOD1 is misfolded, we used the CT4 system to remove oxidized SOD1. We found that removal of the oxidized SOD1 remarkably reduced biomarkers of cellular senescence and increased the proliferation and stemness of NSCs. Extracellular vesicles (EVs) isolated from senescent NSCs contained high levels of oxidized hSOD1 that could accelerate the senescence of young NSCs and induce the death of neurons in the culture. Removal of oxidized hSOD1 on EVs by affinity capture with an interference peptide specific for misfolded SOD1 blocked the EVs-induced senescence of NSCs and death of neurons. Our data demonstrate that oxidized SOD1 is a causing factor of cellular senescence of NSCs. Selective removal of oxidized SOD1 is a promising strategy to rejuvenate NSCs and improve the quality of EVs.

## Methods

### Animals, primary neural stem cell culture and differentiation

Transgenic mice carrying human G93A mutant SOD1 (B6.Cg-Tg(SOD1*G93A)1Gur/J; 004435) and wild-type human SOD1 (B6.Cg-Tg(SOD1)2Gur/J; 002298) were obtained from the Jackson Laboratory. The strains were maintained by breeding hemizygous carrier males to female mice with a C57BL/6 background. Colonies are maintained in the Central Animal Care Services, University of Manitoba.

All animal experiments followed the Guide of Care and Use of Experimental Animals of the Canadian Council on Animal Care. The animal protocol (B2020-027), titled “Post-translational oxidation of SOD1 as a biomarker for aging,” was approved by the Bannatyne Campus Animal Care Committee of the University of Manitoba, whose standards align with the Animal Research: Reporting of In Vivo Experiments (ARRIVE) guidelines 2.0. As previously described [19], timed pregnant female C57BL/6 mice mated with transgenic male mice carrying the full-length wild-type human SOD1 (*hSOD1*^*WT*^), the G93A mutation of human SOD1 (*hSOD1*^*G93A*^) gene or the non-transgenic littermates were humanely euthanized by CO_2_ gas followed by cervical dislocation, and embryos were removed. The day of the positive vaginal plug was considered as E0.5 of pregnancy. The embryos were collected at E14.5 and genotyped by PCR of DNA obtained from mouse tails for the presence of the full-length human SOD1 gene. The brain was removed, and cortices were dissected out, triturated and then passed through a 40 μM strainer. Cells were maintained in Complete StemPro NSC SFM (A1050901, ThermoFisher, Waltham, MA, USA) to form neurospheres in 5–7 days. When the neurospheres were 150–200 μm in diameter, they were dissociated for subculture. For differentiation, the NSCs were harvested by centrifugation and plated on poly-L-ornithine and laminin-coated 6-well plates in Complete StemPro NSC SFM. The enriched neuronal culture was obtained by switching the culture medium to neurobasal/B27 (A3653401, ThermoFisher, Waltham, MA, USA) and confirmed by MAP2 immunostaining.

### Knockdown of misfolded hSOD1 in NSCs by CT4 in vitro and AAV-CT4 in vivo

**Fig. 1:**
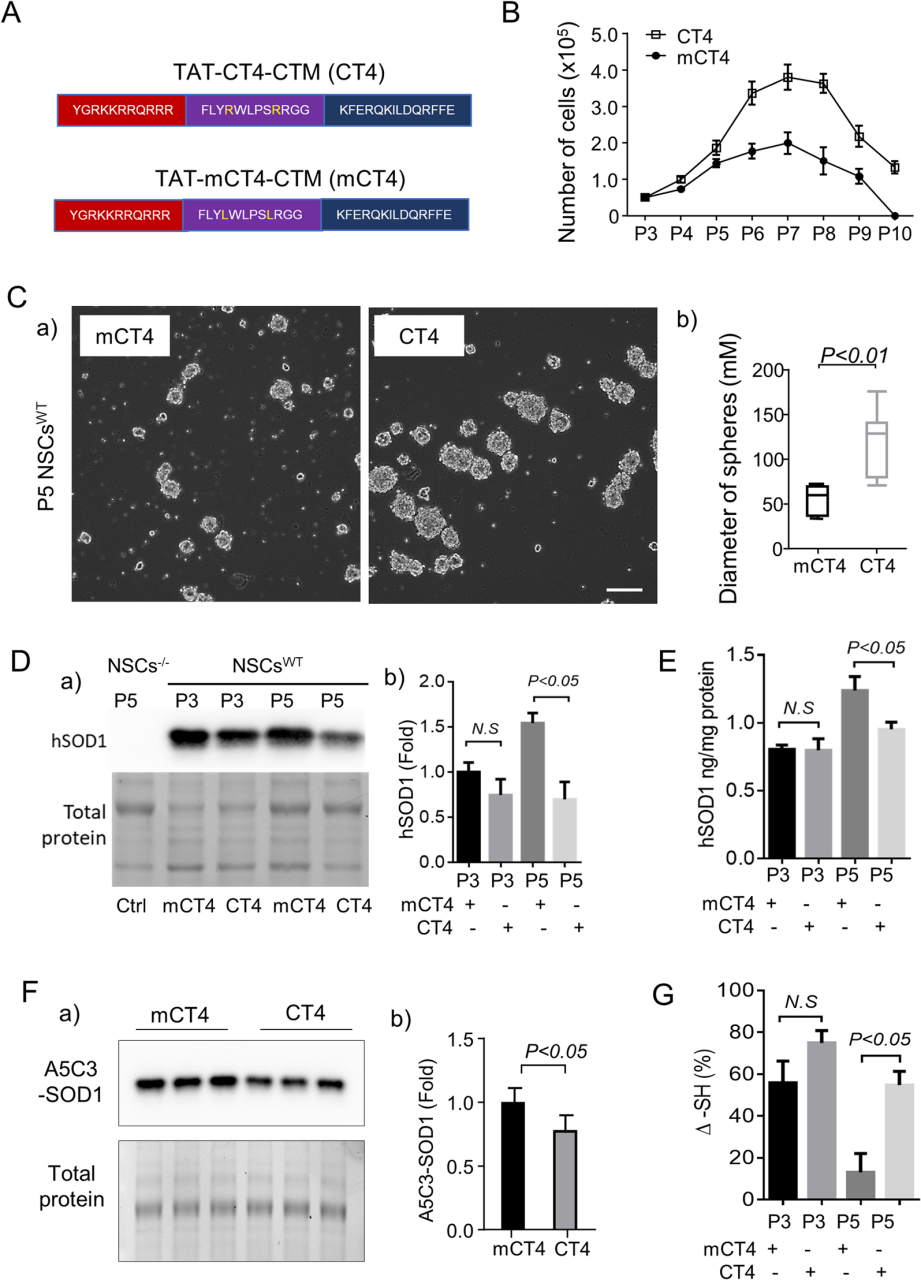
Cellular senescence of NSCs^WT^ after repeated passages in culture. **A.** Sequences of CT4 and mCT4 peptides. **B.** Number of NSCs^WT^ after each passage. CT4 at 5 μM once every other day increased the number of NSCs at every passage. **C.** CT4 doubled the size of neurospheres at P5. a), representative image, scale bar: 100 μm. b) quantification of the diameter of spheres. **D.** Levels of hSOD1 detected by western blot in NSCs^WT^ treated with CT4 or mCT4. Full-length blots are presented in Additional file 1: Fig. 1D. a) gel image; b) densitometry. **E.** Levels of hSOD1 detected by ELISA in NSCs^WT^ treated with CT4 or mCT4. **F.** Levels of misfolded hSOD1 detected by Western blot. Full-length blots are presented in Additional file 1: Fig. 1F. a) gel image; b) densitometry. **G.** Thiol-disulfide status of the hSOD1 in NSCs^WT^ treated with CT4 or mCT4 at P3 and P5. The thiol content was normalized to total hSOD1. For **C** and **F**, Student’s t-test. Two-way ANOVA followed by Sidak’s multiple comparisons test was performed for **D**, **E** and **G**. *P < 0.05; **P < 0.01. All analysis was done in triplicate from three independent cultures

Selective knockdown of misfolded hSOD1 was achieved through a peptide-directed chaperone-mediated approach, as described previously [18]. Briefly, the CT4 fusion peptide consists of the cell penetration domain of the HIV TAT protein, the CT4 sequence that selectively binds to misfolded hSOD1, and the CMA targeting motif (CTM) that is destined for lysosomes [20] (Fig. 1A). The fusion peptide binds specifically to the derlin-1 binding region (DBR) of hSOD1, which is exposed only when hSOD1 is misfolded, and robustly removes misfolded hSOD1 through lysosomal degradation. Knockdown of the misfolded hSOD1 in NSCs was achieved by applying the CT4 fusion peptide to the culture medium at 5 μM every other day. Cell count was done by trypan blue staining, and viable cell numbers were acquired by TC20 automated cell counter from Bio-Rad (Hercules, CA).

For the in vivo study, the CT4-CTM was introduced using the AAV-PHP.eB-CT4 construct. This was achieved by incorporating the CT4-CTM expression cassette into the pUCmini-iCAP-PHP.eB plasmid, kindly provided by Dr. Viviana Gradinaru (Addgene plasmid # 103,005, RRID: Addgene_103005). Following sequence confirmation, the vectors were produced by the Hotchkiss Brain Institute at the University of Calgary. Transgenic hSOD1-G93A mice (n = 5 in control and n = 6 in the AAV-CT4 group) at the age of 100 days received an intravenous injection of 1 × 10^11^ vector genomes (vg) per mouse and were euthanized for analysis at 135 days old.

### CT4-GST pulldown assay of misfolded hSOD1

Direct binding between CT4 and misfolded hSOD1 was assessed using bacterially expressed CT4-Glutathione S-transferase (GST)-fused proteins. Briefly, the derlin1-CT4 epitope (FLYRWLPSRRGG) cloned into the pGEX-4 T-1 vector was transformed into BL21 competent cells. A clone was cultured in LB media containing 50 μg/ml of ampicillin. GST or GST-fused GGGGS-CT4 was expressed at an OD600 of approximately 0.6 with 0.5 mM IPTG. The bacterium pellet was re-suspended in lysis buffer (30 mM Tris–Cl (pH 7.5), 0.1 mM NaCl, 1 mM DTT, 1% NP-40, and protease inhibitors), sonicated and centrifuged at 12,000 rpm for 30 min. The soluble fraction containing GST or GST-GGGGS-CT4 fusion proteins was incubated with glutathione-Sepharose 4B beads (Cytiva, Marlborough, MA, USA) for 1 h to immobilize GST or GST-GGGGS-CT4. To identify direct binding between misfolded hSOD1 and CT4, the immobilized GST-fused proteins were incubated with samples at 4 °C for 2 h. After washing twice with PBS, the bound proteins were separated by SDS-PAGE and detected by immunoblotting.

### Tissue collection and western blot

Mice were deeply anesthetized with isoflurane and transcardially perfused with ice-cold PBS. Lumbar spinal cords were collected and homogenized in 10 volumes of IP lysis buffer (Pierce, Cat#87787, ThermoFisher) with 1%(v/v) protease inhibitor cocktails (Thermo Fisher, #78429). Homogenates were centrifuged at 21,000×*g* for 30 min at 4 °C, and supernatants were collected for analysis. For the cell culture samples, cells were washed with ice-cold PBS, suspended in lysis buffer, and transferred into a microcentrifuge tube. The cell suspensions were maintained in agitation for 30 min at 4 °C. Then, the cell lysates were centrifuged at 21,000×*g* for 30 min at 4 °C, and the supernatants were collected. After BCA assay (Pierce™ BCA Protein Assay Kit, ThermoFisher, Waltham, MA) for protein concentrations, the samples were boiled for 5 min in Laemmli sample buffer containing 2.5% β-mercaptoethanol, separated on 12% TGX Stain-Free polyacrylamide gels (Bio-Rad, Hercules, CA. Cat #1610185), and transferred to PVDF membranes on a Trans-Blot Turbo Transfer System (Bio-Rad, Hercules, CA, USA). Membranes were blocked with 5% (w/v) fat-free dry milk in Tris-buffered saline (10 mM Tris–HCl, pH 7.5, 150 mM NaCl) containing 0.05% Tween-20 for 1 h and incubated with primary antibodies overnight at 4 °C. The primary antibodies used were as follows: anti-hSOD1 (ab52950, RRID: AB_2193891, 1:2000, Abcam, Cambridge, UK), anti-misfolded hSOD1 (A5C3) (MM0070-3-P, 1:200, Medimabs, Montréal, QC, Canada), anti-p53 (ab26, RRID:AB_303198, 1:1000, Abcam, Cambridge, UK) and anti-p16^INK4A^ (sc166760, 1:50, RRID:AB_206006, Santa Cruz, CA, USA). Blots were washed thrice in TBST buffer and then incubated with appropriate secondary antibodies for 1 h at room temperature. The protein bands were visualized with the enhanced chemiluminescence reagent (ECL Prime, RPN2232, GE Healthcare, Chicago, IL, USA) on an imager (ChemiDoc MP, imaging system, Bio-Rad, Hercules, CA, USA).

### Immunocytochemistry and SA-β-gal staining

Cells on coverslips were fixed with 4% paraformaldehyde in PBS pH 7.4 for 15 min at room temperature and then washed three times with PBS. Samples were incubated for 10 min in PBS containing 0.25% Triton X-100 (PBST) to improve the penetration of antibodies. After washing with PBS, the coverslips were incubated in 1% BSA in PBST for 30 min to block the nonspecific binding of the antibodies. After blocking, samples were incubated with primary antibodies in 1% BSA overnight at 4 °C. The primary antibodies used were as follows: anti-SOX2 (ab79351, RRID: AB_10710406, 1:1000, Abcam, Cambridge, UK), anti-Ki67 (9129S, 1:1000, NEB, Ipswich, MA, US), anti-Nestin (sc-33677, RRID: AB_627995, 1:2000, Santa Cruz, CA, USA), anti-HMGB1 (ab228624, RRID: AB_2937009, 1:1000, Abcam, Cambridge, UK), anti-misfolded hSOD1 (B8H10) (MM-0070-P, RRID: AB_2909641, 1:500, Medimabs, Montréal, QC, Canada) and anti-MAP2 (sc-20172, RRID: AB_2250101, 1:2000, Santa Cruz, CA, USA). Following three additional washes, appropriate secondary antibodies were applied and incubated for 1 h at room temperature in the dark. After being washed three final times, cells were incubated with Hoechst 33,342 (Calbiochem, CA, USA) to counterstain for nuclei, and then coverslips were mounted with a drop of mounting medium (Dako North America, Inc. Carpinteria, CA 93013, USA). For SA-β-gal staining, fixed cells were washed with PBS, then 500 μL of β-Galactosidase Staining Solution (9860S, NEB, Ipswich, MA, USA) was added into each well and incubated at 37 °C overnight according to the manufacturer’s instructions. The cells were imaged on a Carl Zeiss AxioImager Z2 microscope with Zen Pro imaging software (Zeiss, Oberkochen, Germany). SA-β-gal staining was quantitated using colour threshold analysis and particle count feature with the Image J version 1.53t (NIH, Bethesda, MD). SA-β-gal staining was also verified with manual counting from 6 random fields per coverslip.

### SOD1 aggregation, thiol oxidation and lactate dehydrogenase (LDH) assay

Differential extractions were used to assess SOD1 aggregation as previously described [21, 22]. Cells were scraped from the culture dish in PBS and centrifuged to pellet the cells before the pellets were re-suspended in 100 μl 1 × TEN (10 mM Tris, 1 mM EDTA, and 100 mM NaCl). The re-suspended cell culture pellets were then mixed with an equal volume of extraction buffer A (1 × TEN, 1% Nonidet P40, and protease inhibitor cocktail 1:100 dilution) and sonicated with a probe sonicator at 50% output for 30 s (50 W at 20 kHz, FB50, Fisher Scientific, Pittsburgh, PA, USA). The resulting lysate was centrifuged for 5 min at > 100,000 × g in an ultracentrifuge (Optimal L-90 k, Beckman Coulter, USA) to separate the pellet (P1) from the supernatant (S1). The supernatant (S1) was decanted and saved for analysis. The pellet (P1) was washed with 200 μl of extraction buffer B (1 × TEN and 0.5% Nonidet P40) by sonication (50% for 30 s). The extract was then centrifuged for 5 min at > 100,000 × g in a Beckman Airfuge to separate a pellet (P2) from the supernatant. The P2 fraction was re-suspended in buffer C (1 × TEN, 0.5% Nonidet P40, 0.25% SDS, and 0.5% deoxycholic acid) by sonication (50% power setting for 30 s) and saved for further analysis. Protein concentration was measured in S1 and P2 fractions by the BCA method as described by the manufacturer (23,227, ThermoFisher, Waltham, MA). For sandwich ELISA, a Human Cu/ZnSOD Matched Antibody Pair Kit (BMS222MST, RRID, AB_10596959, ThermoFisher, Waltham, MA, USA) was used to detect and quantify protein levels of human Cu/ZnSOD in cell lysates following the manufacturer’s protocol. To assess the thiol-disulfide status of the hSOD1, we incubated the aliquots in an aerobic chamber at room temperature overnight to allow oxidation. At the same time, anaerobic preserved samples were used as a reference. The capture antibody (SOD1 P222MS, 2.5 μg/mL, ThermoFisher, Waltham, MA, USA) was adsorbed on wells of an ELISA plate (F96 cert. MaxiSorp™ Immuno-plate: Nunc, Roskilde, Denmark), to which samples containing the same amount of hSOD1 were applied. Maleimide-activated HRP (31485, ThermoFisher, Waltham, MA, USA) was then used to determine the thiol content of the captured SOD1 among groups as the activated HRP presents an available maleimide group that reacts with sulfhydryl-containing SOD1. LDH assay was performed using cell culture supernatants and lysates following the manufacturer’s protocol (ab282925, Abcam, Cambridge, UK). Absorbance was measured on a Synergy Mx multi-mode microplate reader (BioTek, Winooski, Vermont, USA).

### EV isolation and labelling

The medium of cultured NSCs was collected every other day and centrifuged at 1500 g for 10 min at 4 °C followed by 10,000 g for 30 min at 4 °C to remove cells, membranes, and debris. The resulting medium was then filtered through 0.22 μm filters. EVs were isolated using the ExoQuick-TC kit (SBI, Mountain View, California, USA). The EVs were confirmed by transmission electron microscopy (TEM). To label the EVs and track the internalization, we transfected EVs with Texas-red labelled RNA oligonucleotides using an Exo-Fect exosome transfection kit (System Bioscience, Palo Alto, CA, USA) following the manufacturer’s instruction.

### Electron microscopy (EM)

EVs prepared from culture media were fixed in 2% paraformaldehyde, 3% glutaraldehyde and 0.1 M Sorensen’s buffer (pH = 7.4) for 2 h at room temperature. The fixed EVs were ultracentrifuged and washed with 0.1 M Sorensen’s Phosphate Buffer containing 5% sucrose. Fixed vesicles were absorbed onto 200-mesh copper grids (Electron Microscopy Sciences, Hatfield, PA, USA), and stained with 0.5% aqueous uranyl acetate for 30 s and viewed on a transmission electron microscope (Philips CM‐10, Hillsboro, OR, USA).

### Statistical analysis

All manual counts were performed in a blinded manner except for non-objective computational measurements. Data are presented as the mean ± SD. Sample sizes for each experiment are indicated in the method and results. For statistical comparison between the two groups, the Student’s t-test was used. Multiple comparisons were performed by one-way ANOVA followed by Bonferroni’s Multiple Comparison test or two-way ANOVA followed by Sidak’s multiple comparisons test. Differences were considered significant when *P* < 0.05.

## Results

### Oxidized SOD1 accumulates in NSCs expressing WT hSOD1 (NSCs^WT^) after repeated passages in culture

Previously, we reported that oxidation of hSOD1 occurred at its cysteine 111 residue [10, 12]. Once oxidized, hSOD1 undergoes aberrant misfolding. In the present study, we first determined the expression of misfolded hSOD1 in NSCs^WT^ after repeated passages, a model of NSC senescence. NSCs^WT^ were prepared from transgenic mice expressing the full length of human wild-type SOD1. NSCs isolated from non-transgenic mice (NSC^−/−^) were used as controls. The cells were maintained in complete StemPro NSC media to form neurospheres and then subcultured at a 1:3 ratio every 2–3 days. Cells from passage 3 (P3) were used for experiments. Knockdown of misfolded hSOD1 was achieved with the application of CT4 peptide, which selectively binds to the misfolded hSOD1, facilitating its targeting and subsequent degradation via the lysosomal pathway (Fig. 1A). The CT4 fusion peptide was applied to NSCs for selective knockdown of misfolded hSOD1. At the same time, mCT4, a mutant form of CT4, was used as a control. As shown in Fig. 1B, NSCs^WT^ treated with the control peptide mCT4 kept proliferating after each passage until P7 and the proliferation stopped from P8. Most of the cells could hardly form neurospheres and died at P10. Supplement of CT4 at 5 μM in the culture medium once every other day remarkably increased the proliferation and viability of NSCs^WT^ at all passages compared to those treated with mCT4. Noticeably, CT4 doubled the number of NSCs at P7 and P8.

In the cultured environment, NSCs derived from mice can typically be passaged up to 15 [23]. However, the NSCs that carry hSOD1 cannot be maintained for more than passage 10 (Fig. 1B). This observation led us to hypothesize that the proteotoxicity of hSOD1 might contribute to the early onset of senescence in these cells. Supporting this hypothesis, selective knockdown of misfolded hSOD1 mitigates this premature senescence. This finding aligns with a previous study using mouse-derived NSCs to show that the senescence marker SA-β-gal activity is low at passage 6 but increases sharply, reaching a plateau by passage 10 [24].

Meanwhile, the number of neurospheres formed by NSCs^WT^ started to decline at P3 and became significantly reduced at P5. Treatment with CT4 significantly increased the number and size of neurospheres, an indicator of the stemness of NSCs. At P5, the neurospheres treated with CT4 were twice larger than those treated with mCT4 (Fig. 1C).

From P3 and P5, there was a general increase in the expression of total hSOD1. In P5 cells, treatment with CT4 resulted in lower levels of hSOD1 expression than in mCT4-treated cells. The data were validated by western blot with an antibody (AB52950, Abcam, Cambridge, UK) specific to hSOD1 (Fig. 1D) and an ELISA assay utilizing a Cu/ZnSOD Human Matched Antibody Pair (BMS222MST, ThermoFisher, Waltham, MA) (Fig. 1E). We further conducted a western blot analysis using the A5C3 antibody (MM0070-3-P, Medimabs) specific for misfolded SOD1. As expected, accumulation of misfolded hSOD1 was found in P5 NSCs^WT^, which can be reduced by CT4 significantly (Fig. 1F). Misfolding of SOD1 can lead to the formation of unstable intermediates that are more prone to oxidation. Human SOD1 cysteine residues are particularly susceptible to oxidation and change in the thiol redox state. To test the level of oxidized thiols in hSOD1, cell lysates from P3 and P5 NSCs^WT^ were incubated in an aerobic humidity chamber at 4 °C to allow air oxidation. A second sample set was kept in an anaerobic humidity chamber at 4 °C. These two sets of lysates were then transferred to the hSOD1 antibody (SOD1 P222MS, 2.5 μg/mL)-coated plate to allow hSOD1 immobilization. Maleimide-activated HRP (31,485, ThermoFisher, Waltham, MA, USA) was used to determine the thiol content of the captured SOD1 after that. The signal from this modified ELISA represents the available maleimide group that can react with sulfhydryl-containing SOD1 molecules. A change in reactive thiol content (Δ-SH) was calculated by comparing the percentages in OD450 between the aerobic and anaerobic samples. The amount of oxidized SOD1 is inversely correlated with Δ-SH. As shown in Fig. 1G, there was a significant reduction in Δ-SH between P3 and P5 NSCs^WT^ treated with the mCT4. Targeted removal of misfolded hSOD1 with CT4 significantly reduced the level of oxidized thiols at P5.

### Knockdown of misfolded SOD1 prevents oxidized SOD1 accumulation in NSCs^WT^ and ameliorates senescence-associated phenotypes

**Fig. 2:**
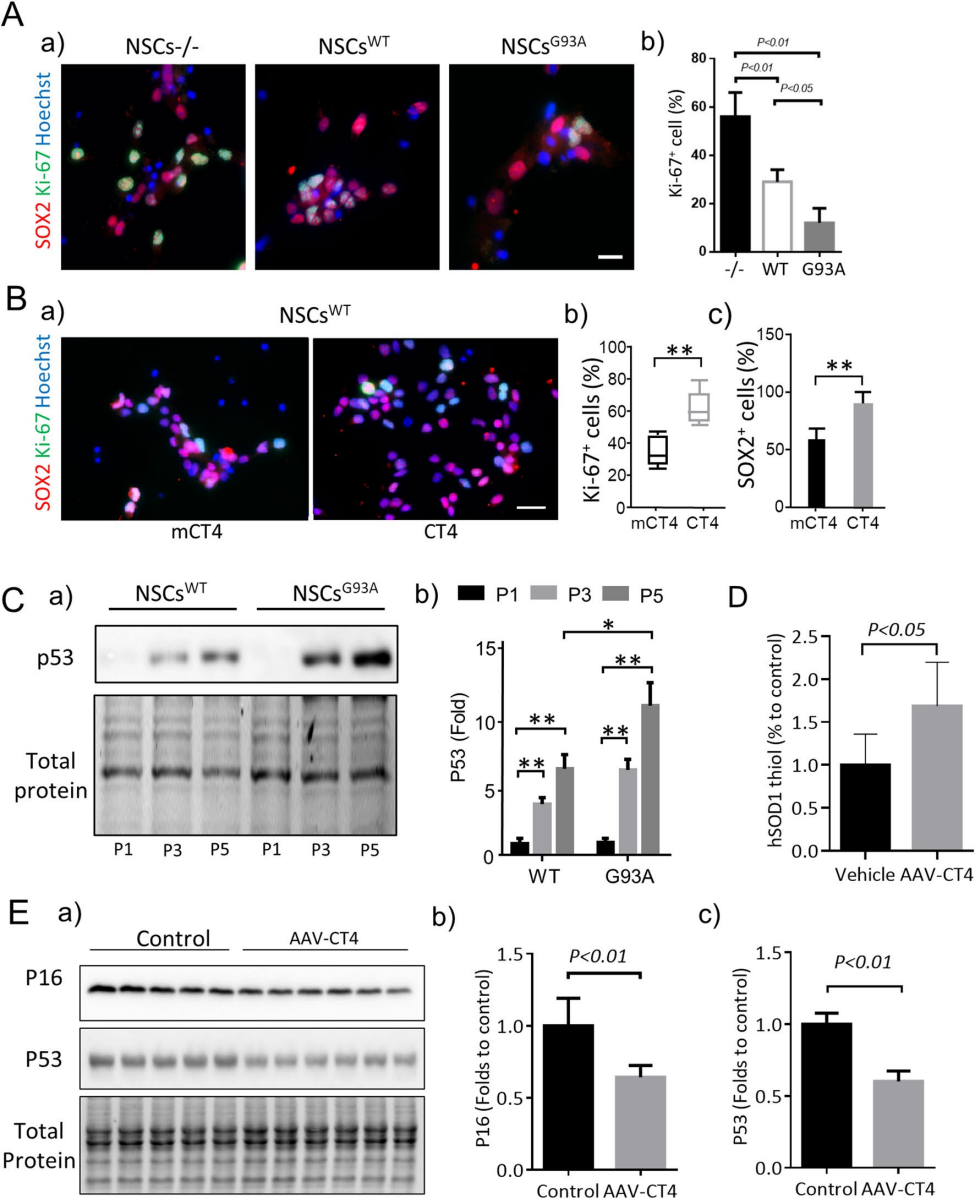
NSCs undergo early entrance into replicative senescence in the presence of misfolded hSOD**. A.** Expression of the WT hSOD1 and the hSOD1 G93A mutation in NSCs results in diminished proliferation. Cell proliferation was visualized by immunostaining with Ki67 (Green) and neural stem cell marker SOX2 (Red) in P5 NSCs^WT^. a) fluorescence image, scale bar: 20 μm. b) quantification of Ki-67 positive cell count. One-way ANOVA followed by Bonferroni’s Multiple Comparison Post-Test. **B.** P5 NSCs^WT^ were treated with mCT4 and CT4 for 24 h, and cell proliferation was detected by immunostaining with antibodies to Ki67 (Green) and to the neural stem cell marker SOX2 (Red). a) fluorescence image, scale bar: 50 μm. Quantification of b) Ki-67 and c) SOX2 + positive cell count. Student’s t-test. **C.** Western blot detection of P53 at different passages of NSCs^WT^ and NSCs^G93A^. Full-length blots are presented in Additional file 1: Fig. 2C. a) gel image; b) densitometry. Two-way ANOVA followed by Sidak’s multiple comparisons test was performed. All in vitro analysis was done in triplicate from three independent cultures. **D.** Quantitative detection of hSOD1 thiol in the spinal cord of the WT hSOD1 mice at 135 days of age. Student’s t-test. **E.** a) Representative immunoblot and quantitative data of b) P16 and c) P53 expression in the spinal cord of the WT hSOD1 mice (n = 5 in control, and n = 6 in AAV-CT4), Student’s t-test. Full-length blots are presented in Additional file 1: Fig. 2E

We then measured the proliferation and self-renewal of NSCs immunologically with antibodies to Ki67 and SOX2 at P5, approximately 2 weeks of culture in vitro. As shown in Fig. 2A, about 58% of passage-matched non-transgenic NSCs (NSCs^−/−^) were positive for Ki67 at P5. Only 30% of cells were positive for Ki67 in the NSCs^WT^, a significant decrease of 51.8% compared to the NSCs^−/−^. These results confirmed that cells with hSOD1 transgene exhibited proteotoxicity induced by overexpression of hSOD1 and the formation of misfolded and oxidized hSOD1 species, which would contribute to senescence. As a positive control, we included passage-matched NSCs expressing the G93A mutation of hSOD1 (NSCs^G93A^, obtained from G93A-hSOD1 mouse E14 embryos). In the hSOD1^G93A^ group, cells positive for Ki67 were further reduced to 12.4% at P5. In NSCs^WT^ at P5, supplementing with CT4 at 5 μM once every other day to remove misfolded hSOD1 doubled the number of Ki67-positive cells and significantly increased SOX2-positive cells (Fig. 2B). We further analyzed the expression of the protein P53, a marker of cellular senescence, in whole-cell lysates of NSCs^WT^ from passages 1, 3 and 5. While NSCs^−/−^ didn’t express P53 throughout the 5 passages (data not shown), NSCs^WT^ and NSCs^G93A^ showed upregulation of P53 after each passage. Specifically, in the NSCs^WT^ group, P53 increased 4.3-fold at P3 and 7.3-fold at P5 compared to P1. In the NSCs^G93A^, levels of P53 increased by 6.8-fold in P3 and nearly 12-fold at P5 compared to P1 (Fig. 2C). We then administered AAV-CT4 to hSOD1-G93A mice, each at the age of 100 days, with a dose of 1 × 10^11^ viral genomes (vg). Subsequent analysis of spinal cord tissue from these mice, taken at the presymptomatic stage of 135 days, revealed that the free thiol content in the treated mice increased by 1.7-fold compared to the vehicle control, as indicated in Fig. 2D. Furthermore, treatment with AAV-CT4 led to a notable suppression of P16 and P53 protein expression, with reductions of approximately 35% and 40%, respectively, as shown in Fig. 2E.

### EVs from senescent NSCs contained misfolded and oxidized hSOD1

**Fig. 3:**
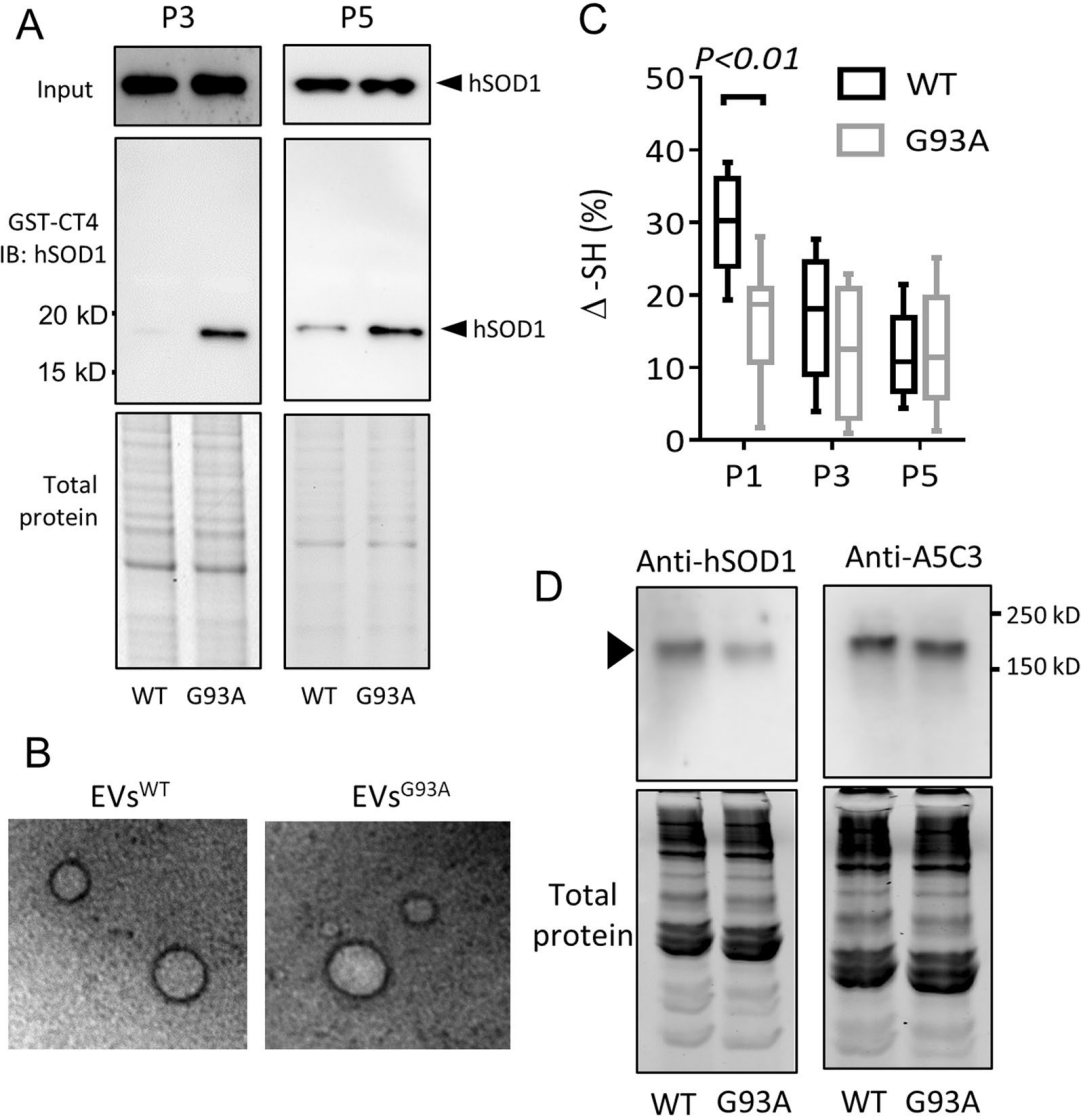
EVs from senescent NSCs^WT^ contain misfolded and oxidized hSOD1**. A.** Level of misfolded hSOD1 in EVs isolated from P3 and P5 NSCs^WT^ and NSCs^G93A^ as detected by affinity binding assay with the GST-tagged CT4. Blots were probed with an anti-hSOD1 antibody. Input: total SOD1 in each lysate. Full-length blots/gels are presented in Additional file 1: Fig. 3A. **B.** EM analysis revealed the presence of EVs by negative staining. Scale bar: 100 nm. **C.** Thiol-disulfide status of the SOD1 in EVs^WT^ and EVs^G93A^ from indicated passages of NSCs. The thiol content was normalized to total hSOD1. Two-way ANOVA followed by Sidak’s multiple comparisons test was performed. **D.** SOD1 expression in EVs from P5 NSCs^WT^ and NSCs^G93A^ was analyzed by non-reducing SDS-PAGE with antibodies to hSOD1 and misfolded hSOD1 (A5C3). Full-length blots are presented in Additional file 1: Fig. 3D. The experiments were done in triplicate from three independent cultures.

Pathogenic hSOD1 is known to propagate through protein secretion and EVs [25]. To determine if senescent NSCs secrete misfolded hSOD1, we prepared EVs from culture media of NSCs carrying *hSOD1*^*WT*^ (EVs^WT^) or *hSOD1*^*G93A*^ (EVs^G93A^) at passages 1, 3, and 5, respectively. Using a GST-CT4 pulldown assay, a band of misfolded hSOD1 was detected in EVs^WT^ at P3. Levels of misfolded hSOD1 in EVs^WT^ increased significantly at P5 (Fig. 3A). At passage 1, there was no detectable misfolded hSOD1 in the EVs^WT^ (Data not shown). Electron microscopy of whole-mount EVs revealed no significant morphological difference between EVs^WT^ and EVs^G93A^ at P5. The EVs had a diameter ranging between 50 and 150 nm (Fig. 3B). To test if misfolded hSOD1 in EVs was oxidized, we aliquoted the EVs lysates and incubated them in an aerobic or anaerobic chamber overnight. The lysates were then added to a plate to immobilize hSOD1. ELISA was used to determine the Δ-SH. As shown in Fig. 3C, a clear decline in Δ-SH was observed in EVs^WT^ across different passages: from 29.7 ± 6.5% at passage 1 (P1) to 17.2 ± 8% at passage 3 (P3) and further down to 12.08 ± 5.8% at passage 5 (P5). This trend suggests a progressive accumulation of oxidized hSOD1 over time. In contrast, EVs^G93A^ displayed a consistently higher level of oxidized SOD1, with Δ-SH values remaining low throughout the passages. This pattern suggests that the EVs^WT^ is comparable to the EVs^G93A^ in terms of hSOD1 oxidation at P5, with the progression occurring incrementally. Using non-reducing SDS-PAGE (-2-Mercaptoethanol/+iodoacetamide), we further confirmed the presence of disulfide cross-linked SOD1 of high molecular weight accumulated in both the P5 EVs^WT^ and EVs^G93A^ (Fig. 3D).

### Exogenous EVs from senescent NSCs induce aging-like phenotypes of young NSCs

**Fig. 4:**
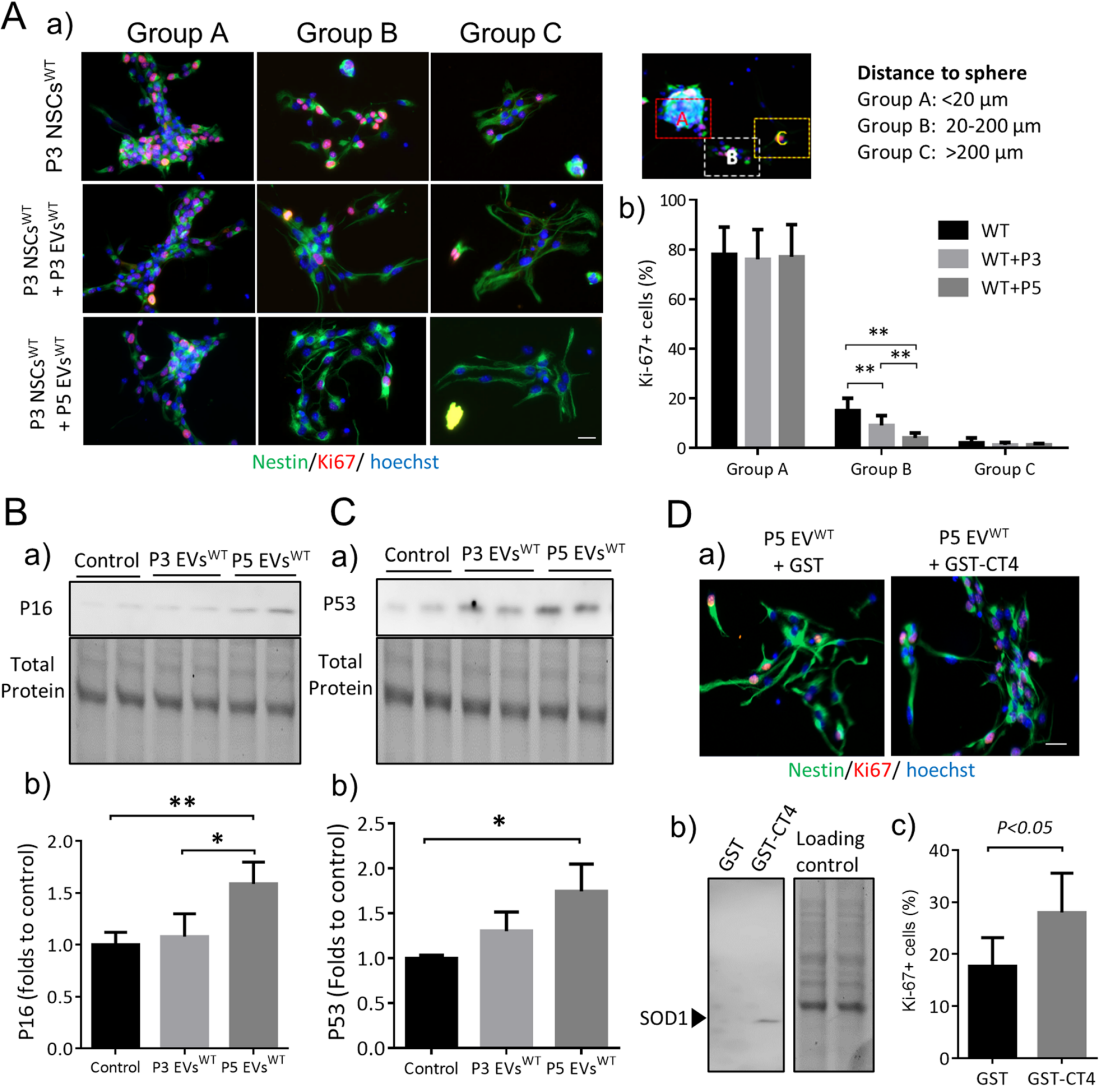
Exogenous EVs from senescent NSCs activate the p53 pathway**. A.** Treatment with P3 and P5 EVs^WT^ diminished proliferation in the recipient P3 NSCs^WT^. Cell proliferation was visualized by a) immunostaining with Ki67 (Red) and Nestin (Green). Scale bar: 20 μm. b) quantification of Ki-67 positive cell count. Two-way ANOVA followed by Sidak’s multiple comparisons test was performed. **B.** and **C.** Expression of P16 and P53 detected by Western blot in P3 NSCs^WT^ treated with P3 or P5 EVs^WT^ for 24 h. Full-length gels are presented in Additional file 1: Figs. 4B and 4C. a) gel images; b) densitometry. One-way ANOVA followed by Bonferroni’s Multiple Comparison Post-Test was performed. **D.** a) Removal of misfolded SOD1 from P5 EVs^WT^ restored the proliferation of NSCs. b) Incubation of GST-tagged CT4 and P5 EVs^WT^ for 2 h resulted in a pulldown of a misfolded SOD1 band as detected by Western blot. After removing misfolded SOD1, P5 EVs^WT^ lost its inhibition on the proliferation of P3 NSCs^WT^ as determined by immunostaining with antibodies to Ki67 (Red) and Nestin (Green). Scale bar: 20 μm. c) For quantitative comparison of Ki-67 positive cells, a Student’s t-test was performed. The experiments were done in triplicate from three independent cultures

Our data above demonstrate that senescent hSOD1-carrying NSCs secrete EVs that contain misfolded and oxidized SOD1. We further investigated the impact of EVs^WT^ from different passages on the proliferation of NSCs. We categorized proliferation patterns of NSCs into three groups based on the distance of Ki67-positive cells from the neurosphere cluster. There were no differences among cells in group A (< 20 μm from neurosphere, which is mainly dividing cells) and group C (> 200 μm, which are primarily migrating cells). In group B, where the cells are 20 μm to 200 μm from the neurosphere, the number of Ki67^+^ cells in P3 NSC^WT^ was significantly reduced from 60% in P3 EVs^WT^-treated cells to 26.7% in P5 EVs^WT^-treated cells as compared to the control group (Fig. 4A). P16, a cyclin-dependent kinase inhibitor that promotes G1 cell cycle arrest, was significantly increased by 1.6-fold in the P5 EVs^WT^-treated cells compared to the non-treated control. In contrast, cells treated with P3 EVs^WT^ didn’t show a significant difference (Fig. 4B). Upregulation of the aging marker P53 was 1.7-fold in the P5 EVs^WT^-treated cells compared to the control (Fig. 4C). We then tested if removal of misfolded hSOD1 from EVs^WT^ by the GST-CT4 affinity capture would restore proliferation of NSCs. Intact exosomes were incubated with GST-CT4 bound Glutathione Sepharose. The GST-CT4, but not the GST alone, pulled down a SOD1 band from P5 EVs^WT^ (Fig. 4D). After the affinity capture of misfolded SOD1, the P5 EVs^WT^ was applied back to P3 NSCs^WT^. The number of Ki67 + -positive cells was restored to 28 ± 7.5% from 17 ± 5.5% in GST-alone control (Fig. 4D).

**Fig. 5:**
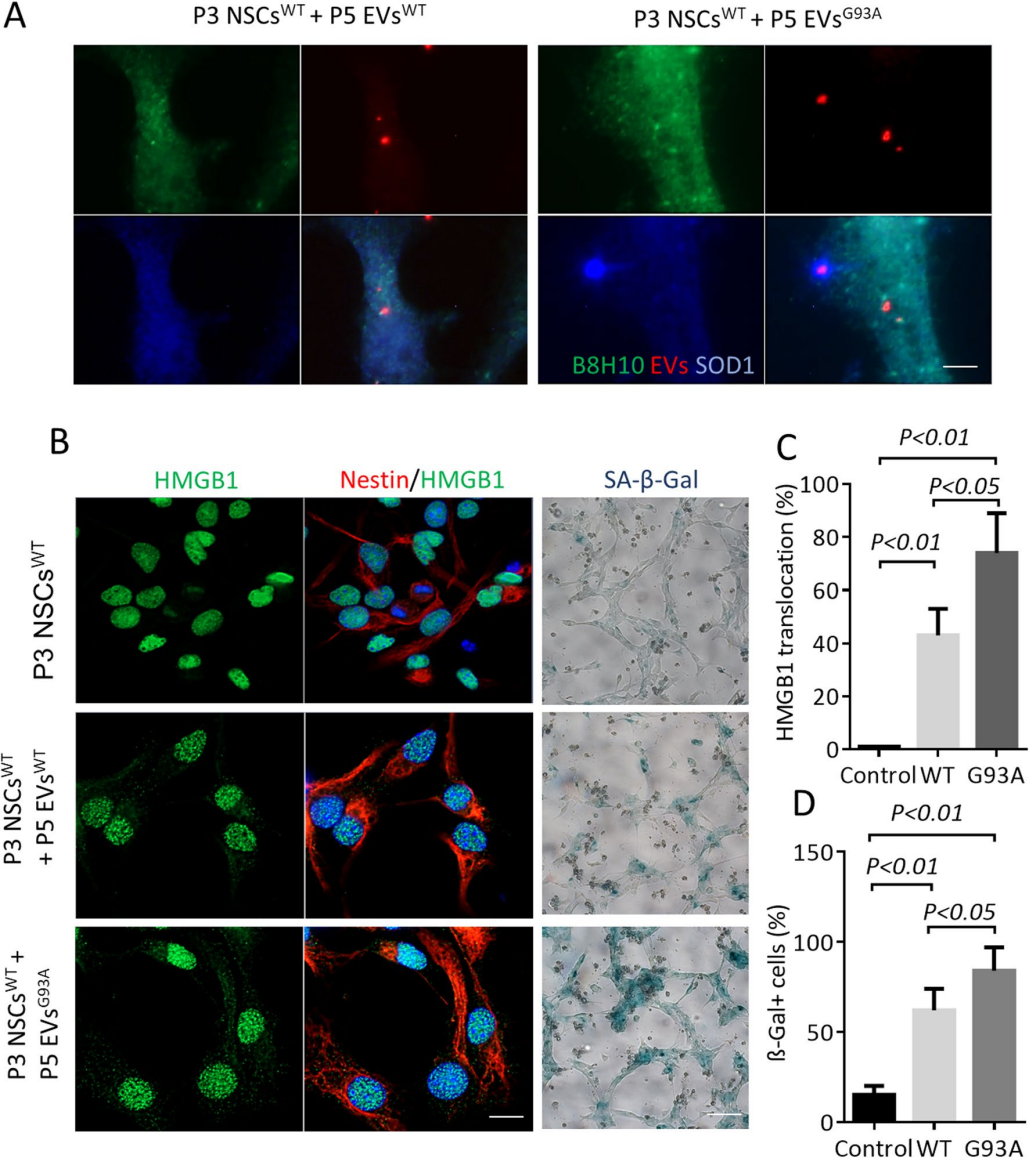
Exogenous EVs from senescent NSCs induce aging-like phenotypes**. A.** Internalization of exogenous EVs. Shown are representative immunofluorescence staining of misfolded hSOD1 (B8H10, Green) and hSOD1 (blue) after treatment with EVs^WT^ and EVs^G93A^ from P5 NSCs. EVs were labeled with Texas Red. Scale bar: 2 μm. **B.** Immunofluorescence staining of P3 NSCs^WT^ with HMGB1 (Green) and Nestin (Red) after treatment with P5 EVs^WT^ and EVs^G93A^. Senescent NSCs were detected by cytoplasmic translocation of HMGB1 and by expressing SA-β-gal. Scale bar: 10 μm**. C.** The translocation of HMGB1 from the nucleus to the cytosol in the P3 recipient NSCs^WT^. **D.** Quantification of SA-β-gal positive cells. For C and D, one-way ANOVA followed by Bonferroni’s Multiple Comparison Post-Test was performed. The experiments were conducted in triplicate from three independent cultures

To further test the effect of EVs from senescent NSCs on young NSCs, EVs^WT^ and EVs^G93A^ at P5 were first labeled with Texas Red and then proportionally applied to P3 NSCs^WT^ for 4 h. Internalization of EVs was detected in the cytoplasm of NSCs^WT^. At 24 h, the labelled EVs^WT^ were co-localized with misfolded hSOD1 that was immunologically detected with the B8H10 antibody (MM-0070-P, Medimabs) (Fig. 5A). Immunocytochemistry revealed cytoplasmic translocation of HMGB1 in 43% of the P3 recipient NSCs^WT^ treated with P5 EVs^WT^. In contrast, in the control group, HMGB1 translocation was found in less than 2% of the cells. The P5 EVs^G93A^ induced an even higher rate (73%) of translocation of HMGB1 in the P3 NSCs^WT^ (Fig. 5B and C). Meanwhile, 58% of P3 NSCs^WT^ treated with P5 EVs^WT^ were positive for senescence-associated β-galactosidase (SA-β-Gal) as compared to 13% in control and 73% in the P5 EVs^G93A^-treated group (Fig. 5B and D).

### Blocking oxidized SOD1 mitigates the neurotoxicity of EVs from senescent NSCs

**Fig. 6:**
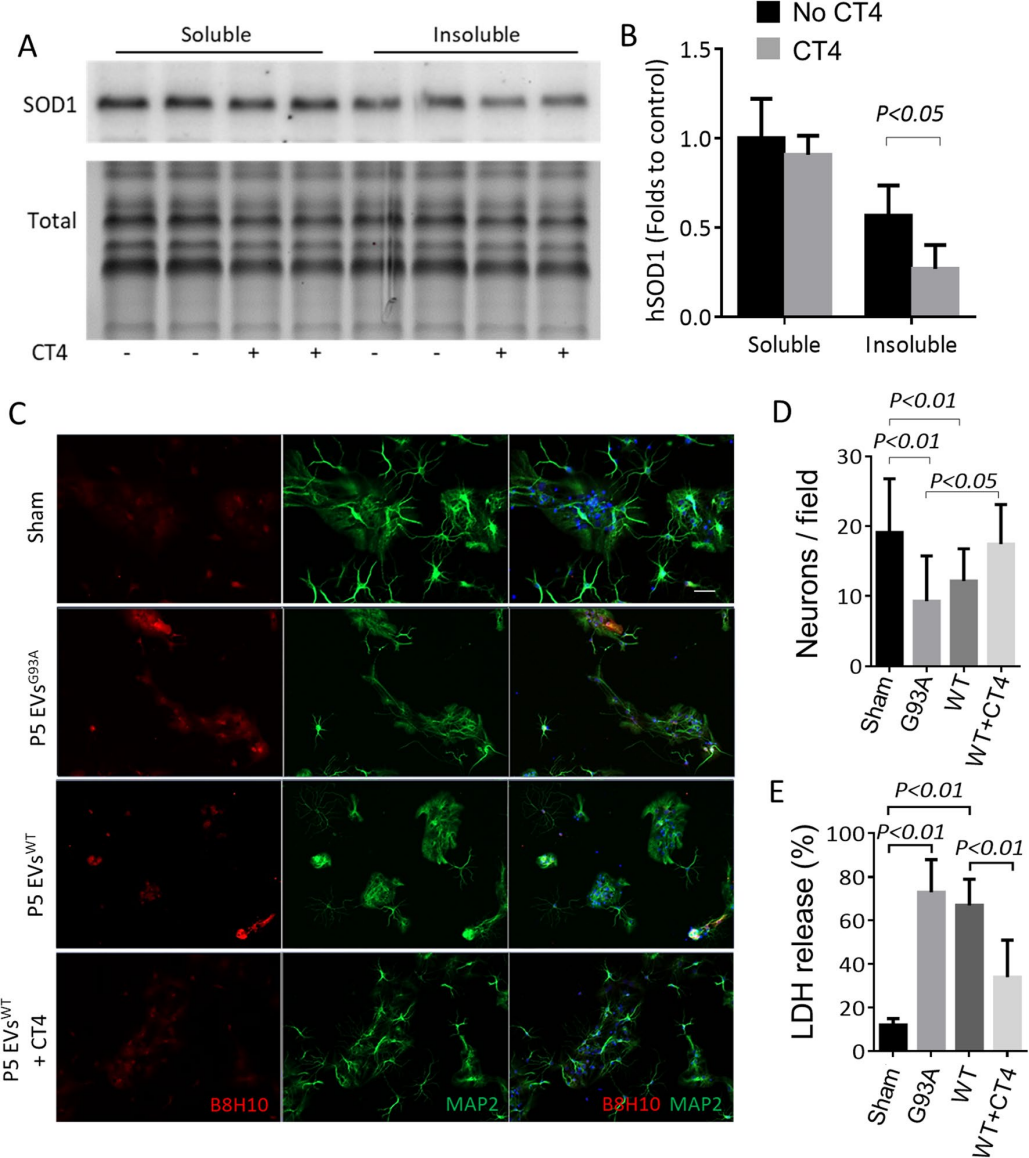
Blocking misfolded SOD1 reduces SOD1 aggregation and mitigates the neurotoxicity of EVs isolated from senescent NSCs. **A.** and **B.** Blocking misfolded SOD1 reduces SOD1 aggregation. P5 EVs^WT^ were incubated for 2 h with or without the CT4 motif before adding it to P3 NSCs^WT^. After 24 h, protein samples were separated into detergent-insoluble and soluble fractions and analyzed by Western blot with an anti-hSOD1 antibody. Full-length blots/gels are presented in Additional file 1: Fig. 6A. Total protein generated by stain-free visualization was used to ensure equal loading. Two-way ANOVA followed by Sidak’s multiple comparisons test was performed. **C.** Neurotoxicity of P5 EVs to P3 NSCs^WT^. Cells were immunostained with antibodies to misfolded hSOD1 (B8H10, Red) and MAP2 (Green). Scale bar: 50 μm**. D.** Quantification of MAP2 positive neurons. **E.** Release of lactate dehydrogenase. For D and E, one-way ANOVA followed by Bonferroni’s Multiple Comparison Post-Test was performed. The experiments were done in triplicate from three independent cultures

As shown in Figs. 1, 2 and 3, NSCs carrying hSOD1^WT^ at P5 exhibited significantly reduced proliferation and stemness, as well as increased expression of senescence-associated biomarkers(Figs. 4 and 5). Therefore, we categorized the P5 hSOD1^WT^ NSCs as senescent stem cells, while the P3 cells were considered young. We conducted further experiments to investigate the effect of CT4 on the toxicity of P5 EVs in neuronal cultures. The isolated P5 EVs^WT^ were incubated for 2 h at 37 °C with the DBR binding motif of CT4 (FLYRWLPSRRGG) to block oxidized SOD1 before they were applied to P3 NSCs^WT^. When oxidized SOD1 was blocked with CT4, the P5 EVs^WT^ induced a 53% decrease of total hSOD1 in the detergent-insoluble fraction of the P3 NSCs^WT^, while in the soluble fraction, it was not significantly decreased at 24 h (Fig. 6, A and B). Next, we cultured the P3 NSCs^WT^ in a differentiation medium and exposed them to P5 EVs^WT^ treated with or without CT4 for 24 h. Immunochemistry confirmed that applying P5 EVs^WT^ induced the B8H10 positive staining in recipient cells, similar to the effects observed with P5 EVs^G93A ^(Fig. 6C). Treatment with P5 EVs^WT^ or EVs^G93A^ resulted in a notable decrease in MAP2-positive neurons, with a 36% reduction observed for EVs^WT^ and a 51% reduction for EVs^G93A^ compared to the sham treatment. However, when P5 EVs^WT^ were pre-treated with CT4, there was an increase in MAP2-positive neurons to an average of 17.45 ± 5.6 neurons/field, in contrast to 12.15 ± 4.6 neurons/field in non-treated P5 EVs^WT^ group, as depicted in Fig. 6D. In addition, we performed a LDH assay at 24 h, corresponding to time points of MAP2 staining using cell culture supernatants and lysates. Treatment with P5 EVs^WT^ or EVs^G93A^ resulted in a significant increase in LDH leakage, reaching approximately a 5.6-fold increase for EVs^WT^ and a sixfold increase for EVs^G93A^ compared to the sham treatment. When P5 EVs^WT^ were pre-treated with CT4, there was a decrease in LDH leakage to an average of 34 ± 17%, in contrast to 67 ± 12% non-treated P5 EVs^WT^ group, as shown in Fig. 6E.

## Discussion

NSCs, especially human NSCs, undergo cellular senescence characterized by irreversible proliferation arrest and loss of stemness after prolonged culture [26]. A growing body of evidence points toward reactive oxygen species as one of the primary determinants of the cellular senescence of NSCs. While compelling correlative data have been generated to support the oxidative stress theory, a direct cause-and-effect relationship between the accumulation of oxidation-mediated damage and cellular senescence of NSCs has not been firmly established [17, 27]. In the cultured environment, mouse NSCs can typically be maintained to 15 passages. The NSCs that carry hSOD1^WT^ can be maintained for no more than 10 passages. We have found that mouse NSCs expressing the full-length human *SOD1* at P5 exhibited significantly reduced proliferation and stemness, as well as increased expression of senescence-associated biomarkers compared to those at P3. Therefore, we categorized the P5 hSOD1^WT^ NSCs as senescent stem cells, while the P3 cells were considered young. In this process, we detected an increase in misfolded human SOD1 and a concomitant rise in the levels of its oxidized form. This suggests that the processes of misfolding and oxidation of SOD1 are interconnected, potentially exacerbating each other, and fostering the formation of aggregates. These aggregates are sequestered into EVs, which may subsequently disseminate these aberrant post-translational modifications to unaltered SOD1 proteins through a prion-like mechanism, perpetuating a cascade of propagation. Selective removal of misfolded hSOD1 from the NSCs remarkably restores the proliferation and stemness of the NSCs. We further show that EVs prepared from culture media of senescent NSCs carrying the wild-type *hSOD1* gene contain a high level of misfolded hSOD1 that is oxidized, as evidenced by the reduction of reactive thiols of hSOD1. Application of the exogenous EVs from senescent NSCs^WT^ to young NSCs accelerates the senescence of the young NSCs. Removal of misfolded hSOD1 from the EVs by affinity capture abolishes its senescence-inducing activity and restores the proliferation of young NSCs. Our data demonstrate that oxidized hSOD1 is a causal factor in the cellular senescence of NSCs.

Aging leads to disrupted cell metabolism and results in the intracellular accumulation of misfolded and aggregated proteins. Protein misfolding is both a cause and a consequence of increased cellular stress. It has been observed in the nervous tissues of patients affected by various age-related disorders, such as Alzheimer’s disease and ALS [28]. The ability of NSCs to self-renew and differentiate into multiple neural lineages makes them indispensable in maintaining a multipotent reservoir and generating differentiated cells. A recent study shows that self-association and subsequent deposition of the misfolded protein aggregates are mainly present in the resting NSCs and the accumulation of protein aggregates contained within lysosomes in healthy young adults [3]. Young stem cells are relatively robust enough to handle misfolded protein through high ATP-dependent TRiC/CCT Chaperonin [29]. In aged NSCs, the ability to reactivate (i.e., re-entry to the cell cycle) is limited due to fewer lysosomes being available to fuse with autophagosomes for autophagy to remove toxic proteins [3]. Defects of lysosomes and accumulation of protein aggregates also display delayed proteotoxicity to differentiated neurons derived from aged NSCs. Manipulation of autophagy to enhance the clearance of protein aggregates has been shown to rejuvenate aged NSCs [7]. Given that NSCs can be isolated from the brains of presymptomatic mice but not symptomatic mice carrying the SOD1 G93A mutation, which are known to accumulate aggregate-prone hSOD1 [30], we would speculate that oxidized SOD1 contributes to the progression of aging by impairing NSCs proteostasis.

ALS-linked hSOD1 variants have been shown to induce a prion-like transmission [31]. Misfolded wild-type hSOD1 is detected in the cerebrospinal fluid (CSF) from patients with sporadic ALS [32], and the CSF samples exhibit significant toxicity toward cells. It remains, however, to be tested whether wild-type hSOD1 secreted in its oxidized form from aged NSCs triggers further misfolding and aggregation of proteins in neighboring cells. Thiol oxidation in SOD1 facilitates its misfolding and contributes to its gain of toxic functions [33]. Human SOD1 has four cysteine residues (Cys6, Cys57, Cys111, and Cys146). An internal disulfide bond exists between Cys57 and Cys146, which contributes to the high stability of the SOD1 protein. At the same time, the cysteine residues at positions 6 and 111 are free. They can be reversibly oxidized to a sulfenic acid (Cys-SOH) by low concentrations of hydrogen peroxide or irreversibly to sulfinic (Cys-SO_2_H)/sulfonic (Cys-SO_3_H) acids by high concentrations of hydrogen peroxide [34–36]. The aging process is associated with a decline in mitochondrial quality control for the breakdown of oxidized proteins within mitochondria. This decline results in the accumulation of oxidized proteins, leading to cellular stress and potentially contributing to aging [37]. Our data show that oxidation of hSOD1 occurs in NSCs after repeated passages in culture. Oxidized SOD1 inevitably undergoes aberrant misfolding [12] and is not readily degraded through common protein degradation pathways. As a result, oxidized and misfolded hSOD1 accumulation is detected in aging and age-related neurodegenerative diseases [16, 38]. Based on our data, it is tempting to speculate that oxidized SOD1 is a causal factor for human aging. The high susceptibility of hSOD1 to oxidization may explain why it is more challenging to grow primary human cells than animal cells in culture. Further research is needed to understand this fully.

Extracellular vesicles (EVs), particularly within adult neurogenic niches, play an integral role in cellular communication and coordination [39]. EVs facilitate the transfer of molecular signals between cells, which is crucial for maintaining brain health and function. Emerging from this understanding is the rapidly evolving field of exosome-based therapies in medicine [40]. Their ability to cross biological barriers and deliver therapeutic agents directly to target cells positions them at the forefront of innovative medical treatments, potentially revolutionizing approaches in neurodegenerative diseases, cancer, and regenerative medicine. The biocompatibility and reduced immunogenicity of these vesicles further underscore their potential as a transformative tool in modern healthcare.

A recent study has revealed that proteins can be selectively incorporated into EVs, a process dependent on the membrane protein LAMP2A. This discovery marks a significant advancement in exosome engineering, particularly in embedding bioactive proteins into EVs through sorting motifs [41]. This method deepens the understanding of exosomal cargo transport and offers exciting prospects for novel therapeutic strategies. Conversely, it should be noted that protein aggregates can be expelled from cells via EVs, serving as a mechanism for disposing of toxic proteins. This is particularly significant in cells with low lysosomal numbers or activity [42]. It is worth noting that when EVs penetrate a target cell, they do not immediately undergo lysosomal degradation but initiates the transfer of specific signal transduction pathway to target cells (43). Our findings indicate that hSOD1 is notably concentrated in extracellular vesicles (EVs), and its misfolding, along with oxidative modifications, plays a key role in propagating cellular senescence. Consequently, this aspect must be carefully considered when utilizing EVs for therapeutic applications to ensure efficacy and safety in potential treatments.

## Conclusions

Oxidized hSOD1 accumulates in senescent NSCs. Knockdown of misfolded SOD1 reduces oxidized hSOD1 and remarkably increases the proliferation and stemness of the NSCs. EVs derived from senescent NSCs contain high levels of oxidized hSOD1 and accelerate the senescence of young NSCs. Blocking oxidized hSOD1 in EVs abolishes their senescence-inducing activity. Based on these data, we conclude that oxidized hSOD1 is a causal factor in the cellular senescence of NSCs. Removal of oxidized hSOD1 is a strategy to rejuvenate NSCs and to improve the quality of EVs derived from senescent cells.

### Supplementary Information

**Additional file 1.**  Images of full-length blots/gels.

## Data Availability

The data and materials supporting this study’s findings are available from the corresponding author upon reasonable request.

## Abbreviations

NSC: Neural stem cell
SOD1: Superoxide dismutase 1
hSOD1: Human SOD1
EV: Extracellular vesicle
ALS: Amyotrophic lateral sclerosis
CMA: Chaperone-mediated autophagy
CTM: CMA targeting motif
hSOD1^WT^: Wild-type human SOD1
hSOD1^G93A^: G93A mutation of hSOD1
DBR: Derlin-1 binding region of SOD1
GST: Glutathione s-transferase
LDH: Lactate dehydrogenase
PBST: PBS containing 0.25% Triton X-100
NSCs^WT^: NSCs carrying the wild-type hSOD1
NSCs^−^^/^^−^: Non-transgenic NSCs
NSCs^G93A^: NSCs carrying the G93A mutation of hSOD1
EVs^WT^: EVs derived from NSCs^WT^
EVs^G93A^: EVs derived from NSCs^G93A^
SA-β-Gal: Senescence-associated β-galactosidase
