# Supplementary figures and images for: Oxidized SOD1 accelerates cellular senescence in neural stem cells
Source: Stem Cell Res Ther. 2024 Feb 27;15:55. doi: 10.1186/s13287-024-03669-5 (PMC10900543; doi:10.1186/s13287-024-03669-5)

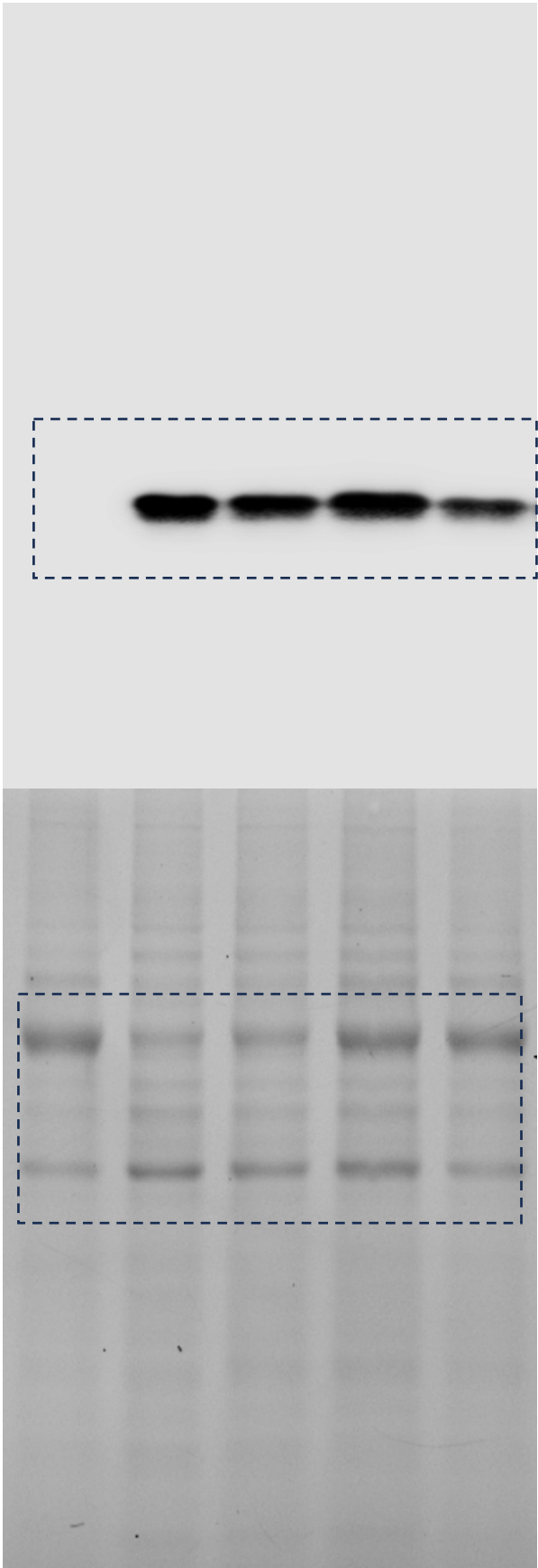

Fig. 1, D

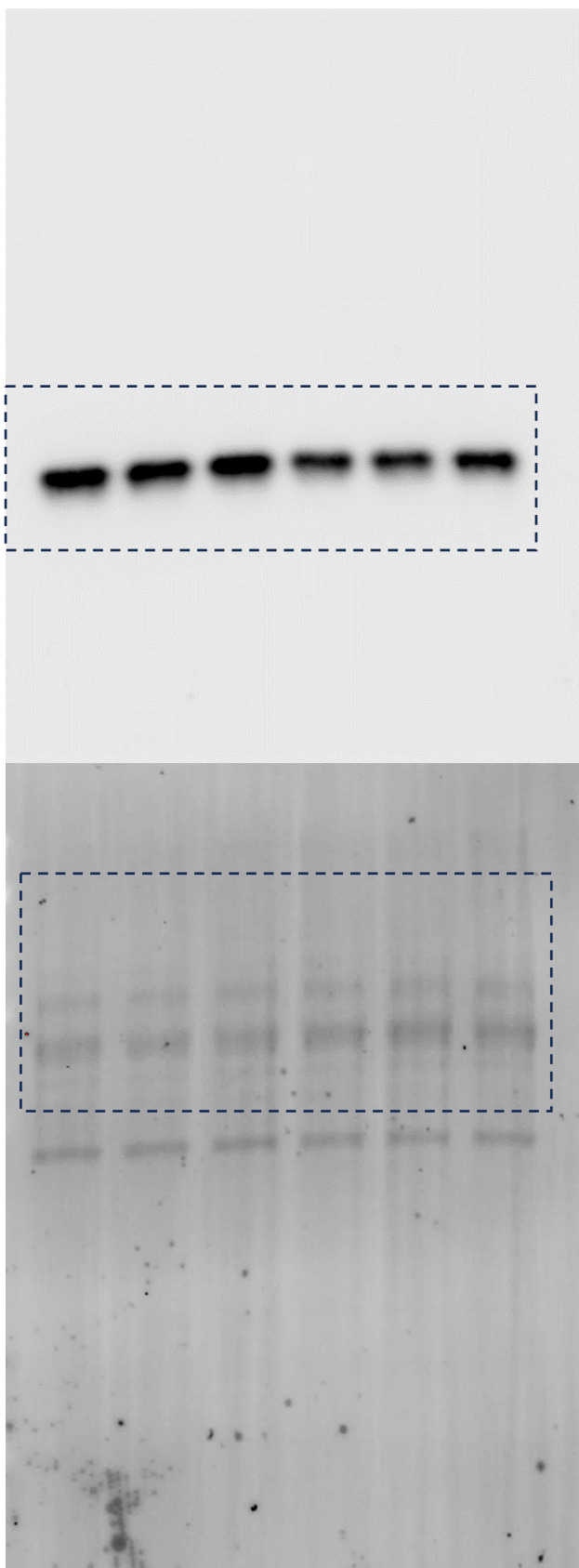

Fig. 1 F

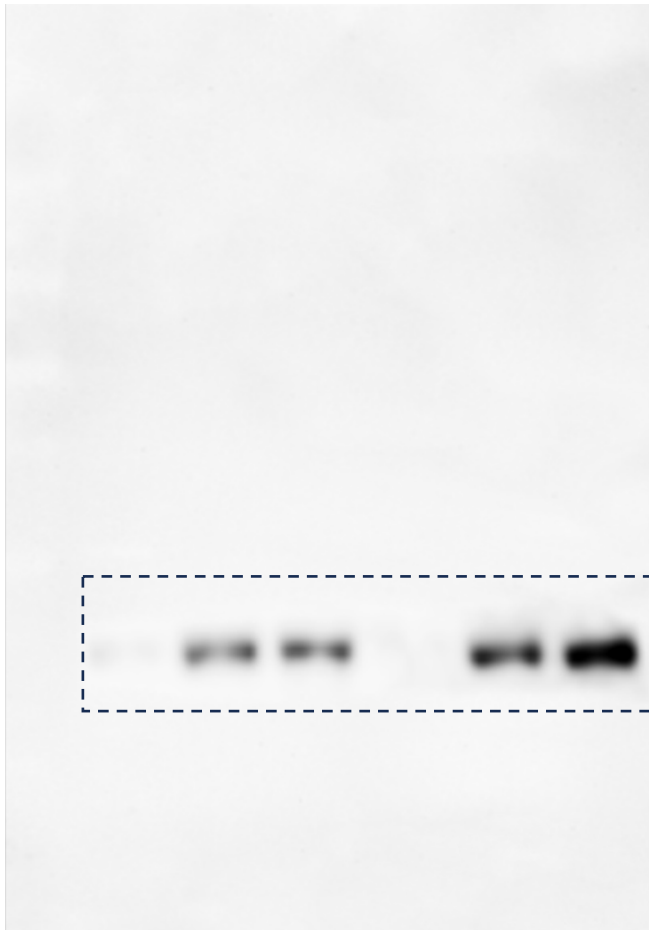

Fig. 2 C

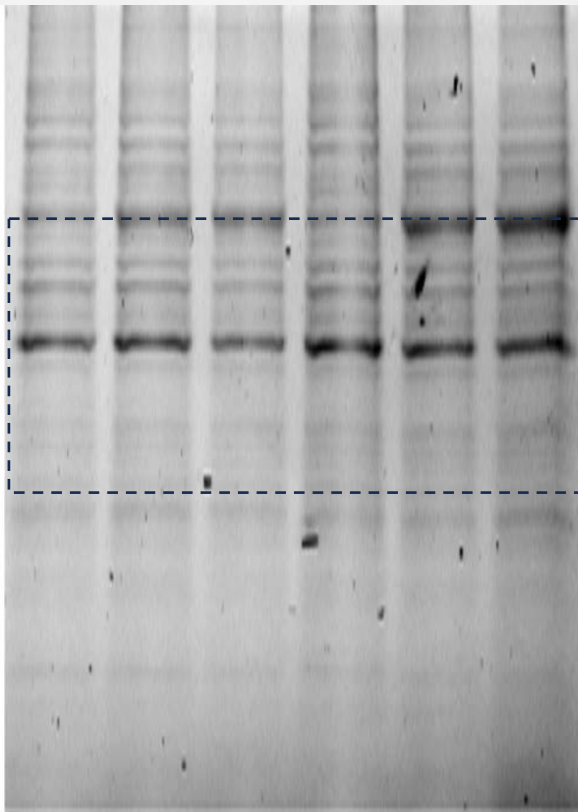

P16

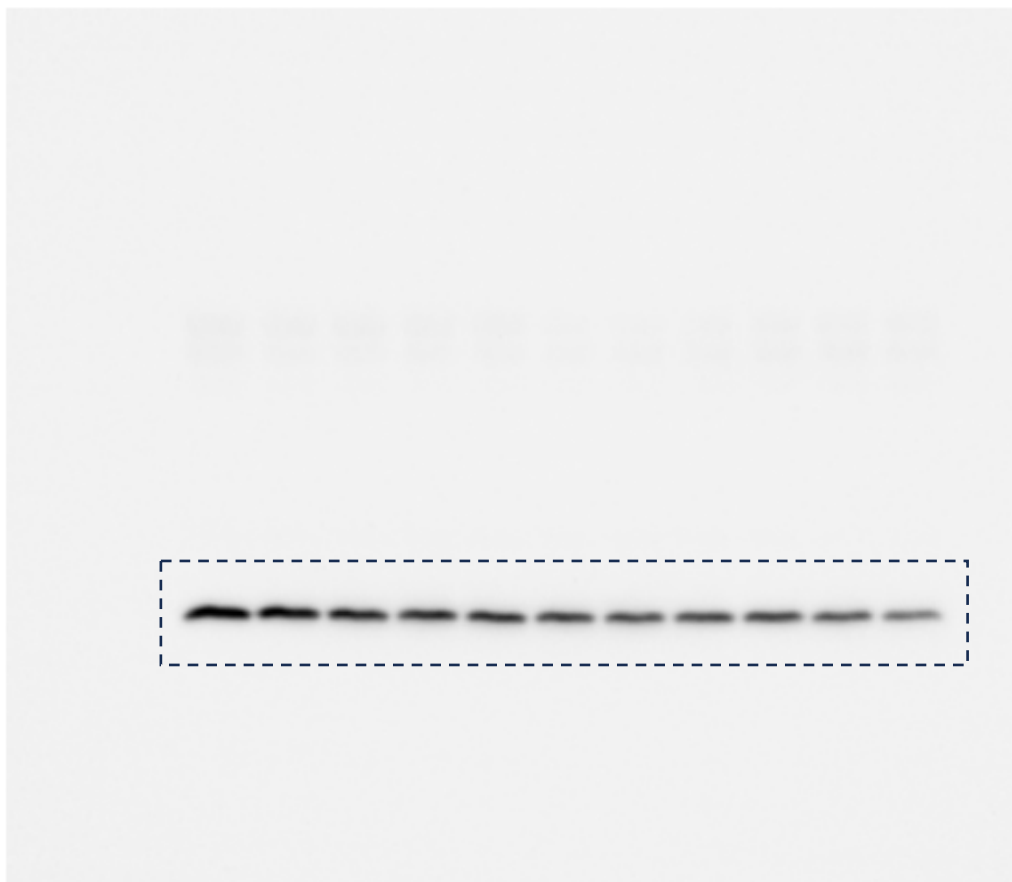

Fig. 2 E

P53

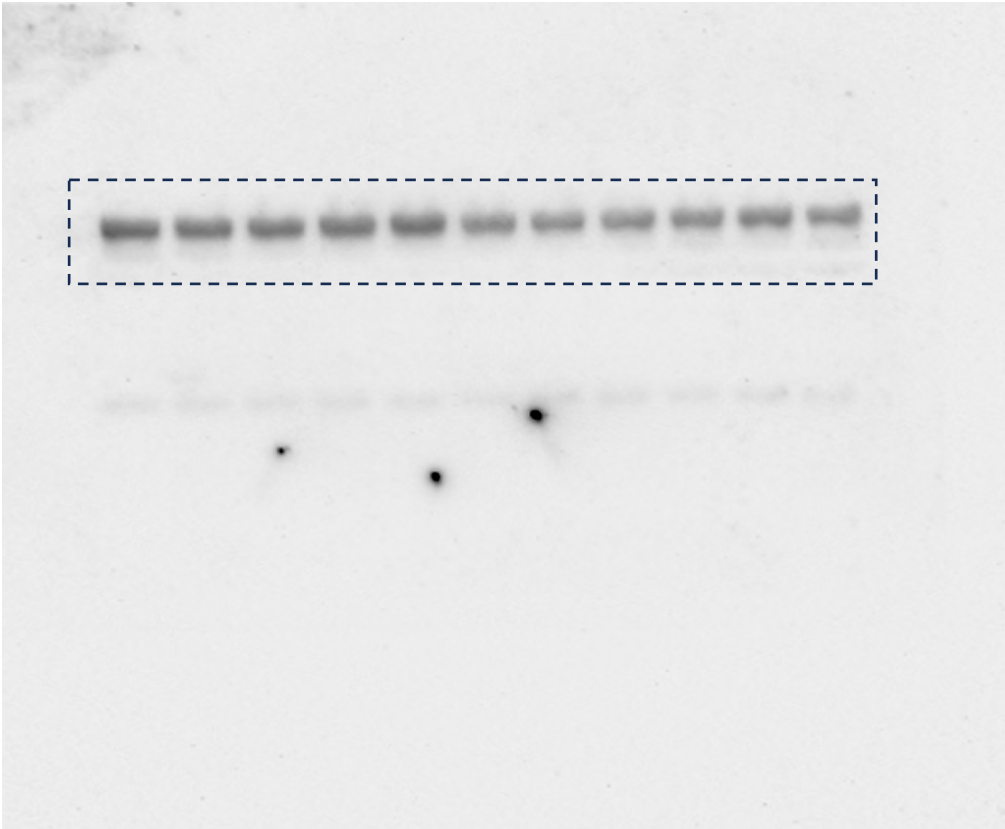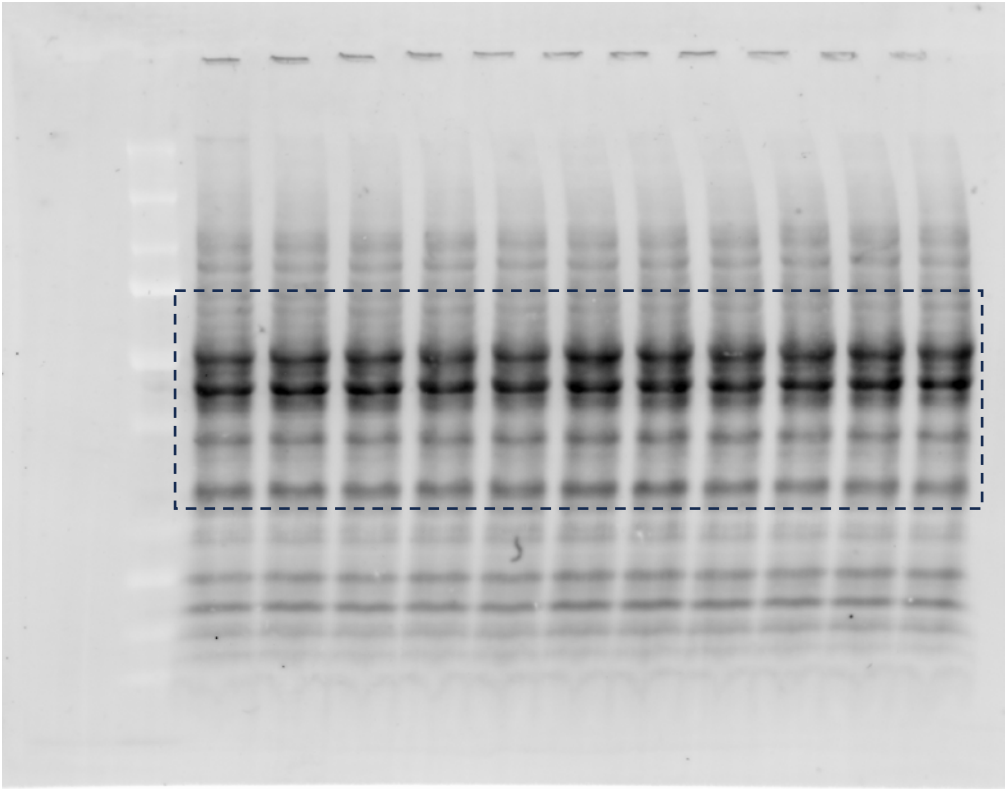

Fig. 2 E

Fig. 3 A

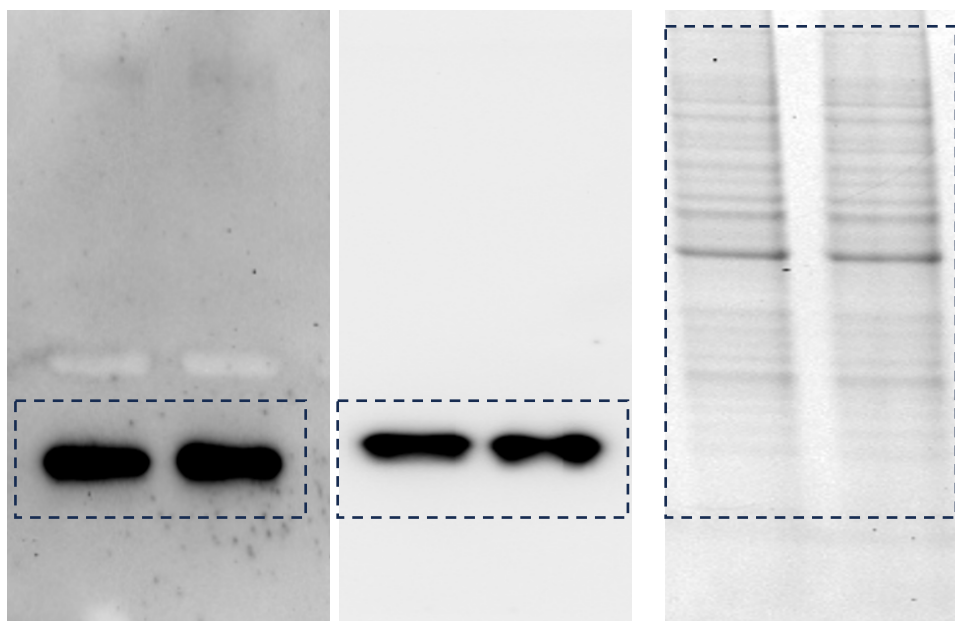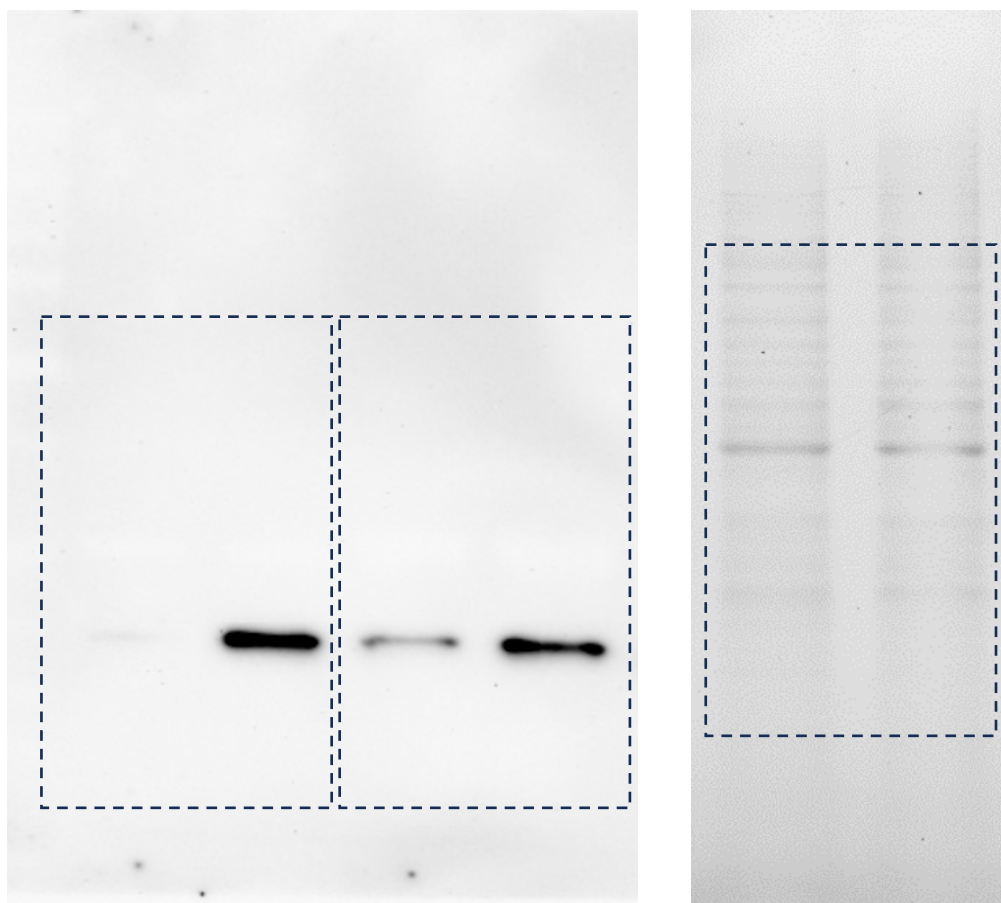

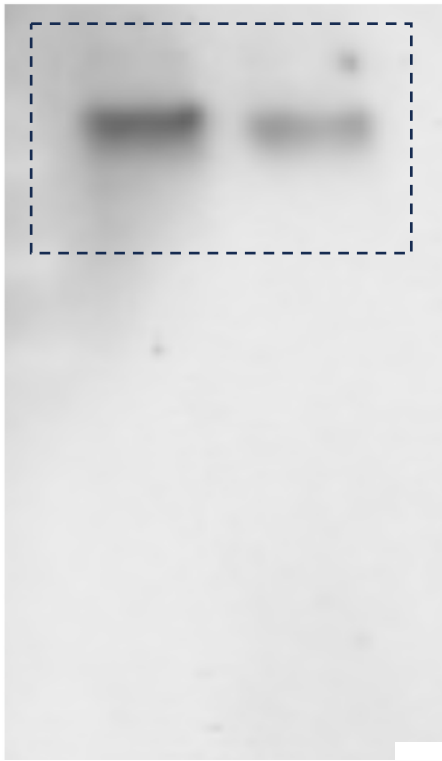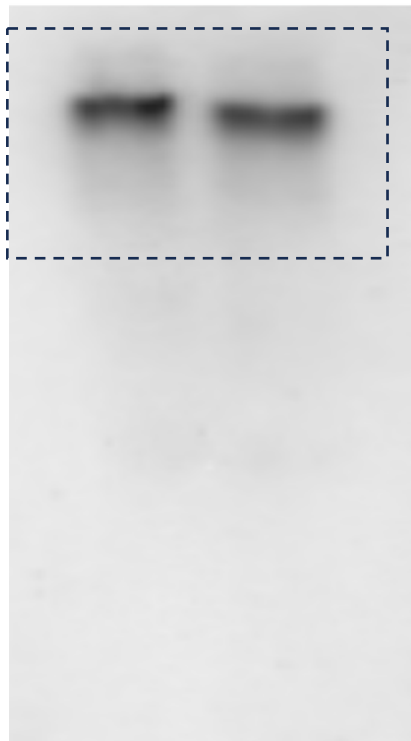

Fig. 3 D

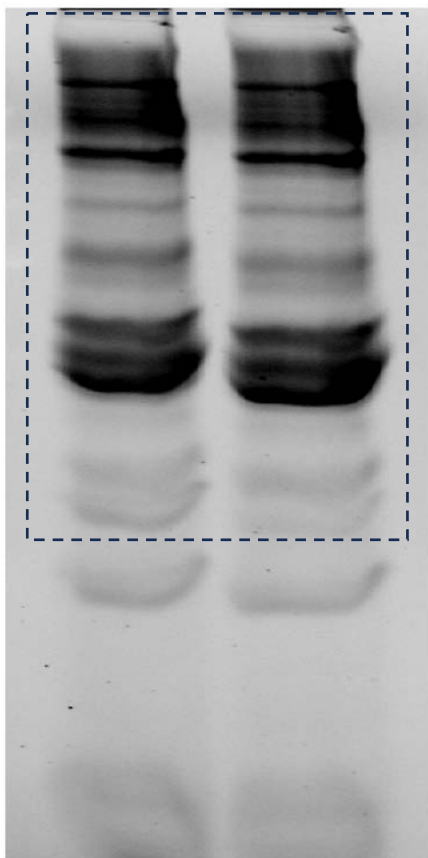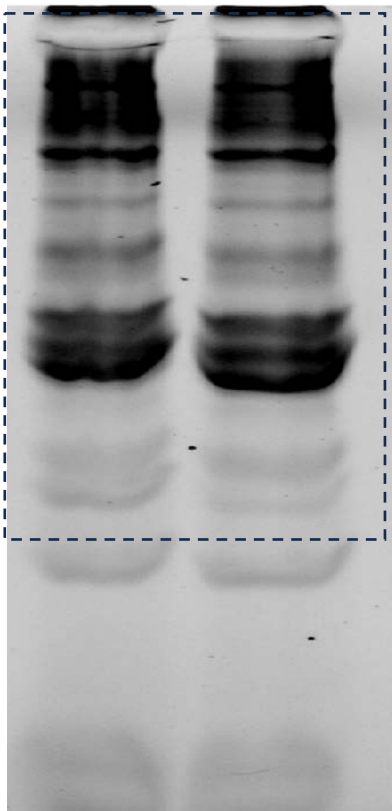

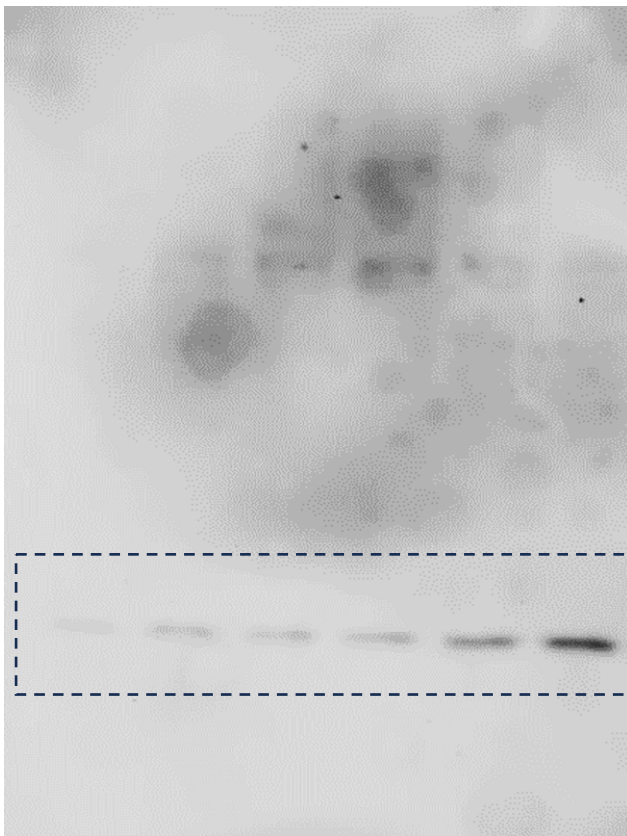

Fig. 4 B

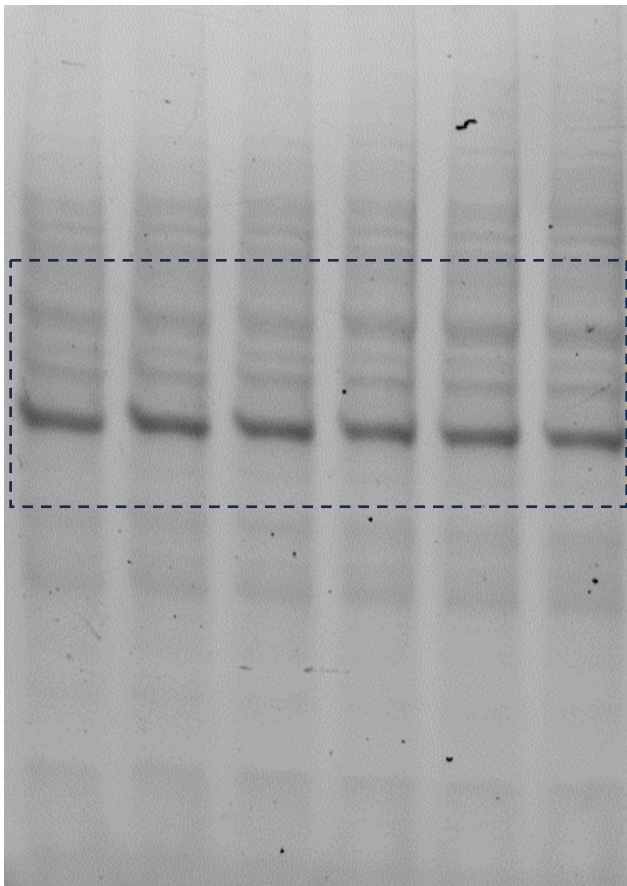

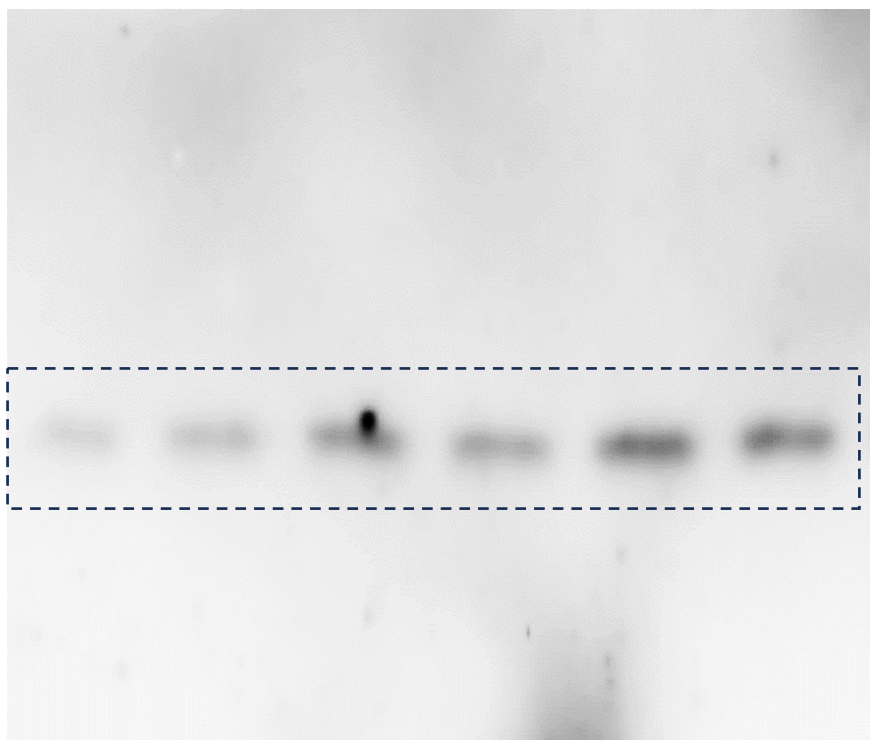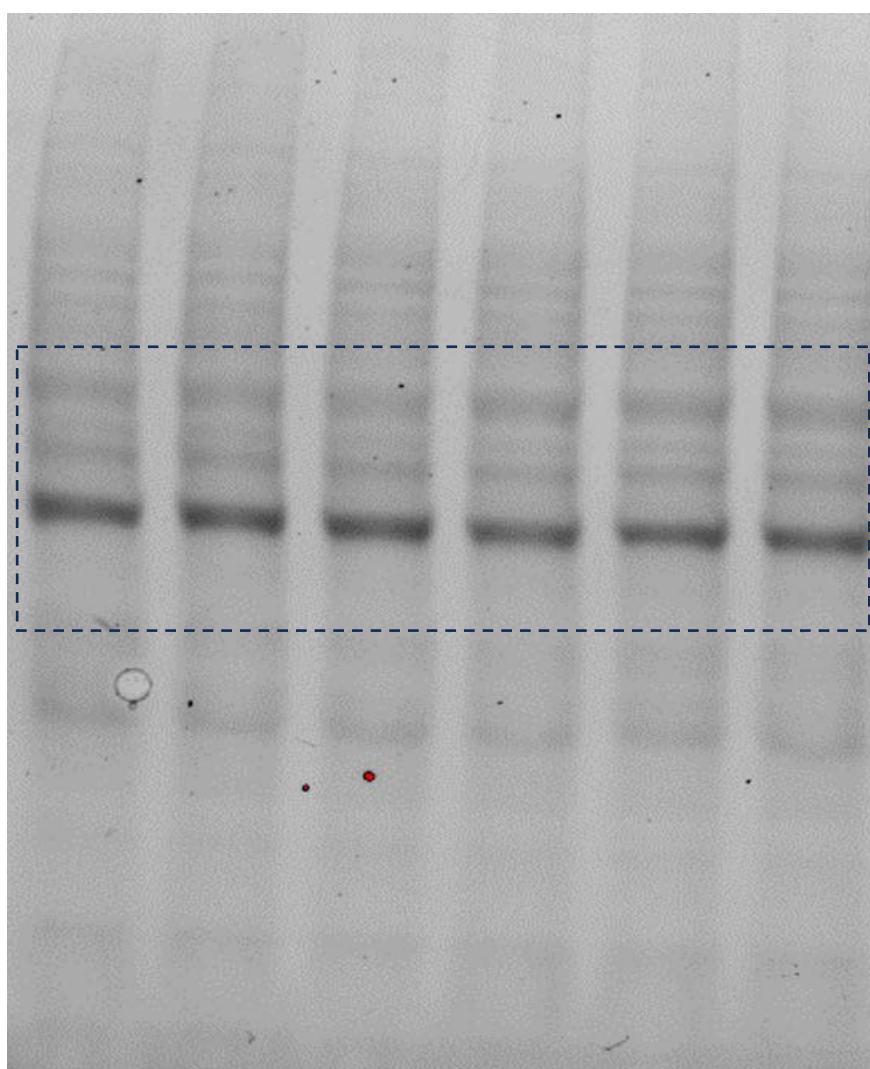

Fig. 4 C

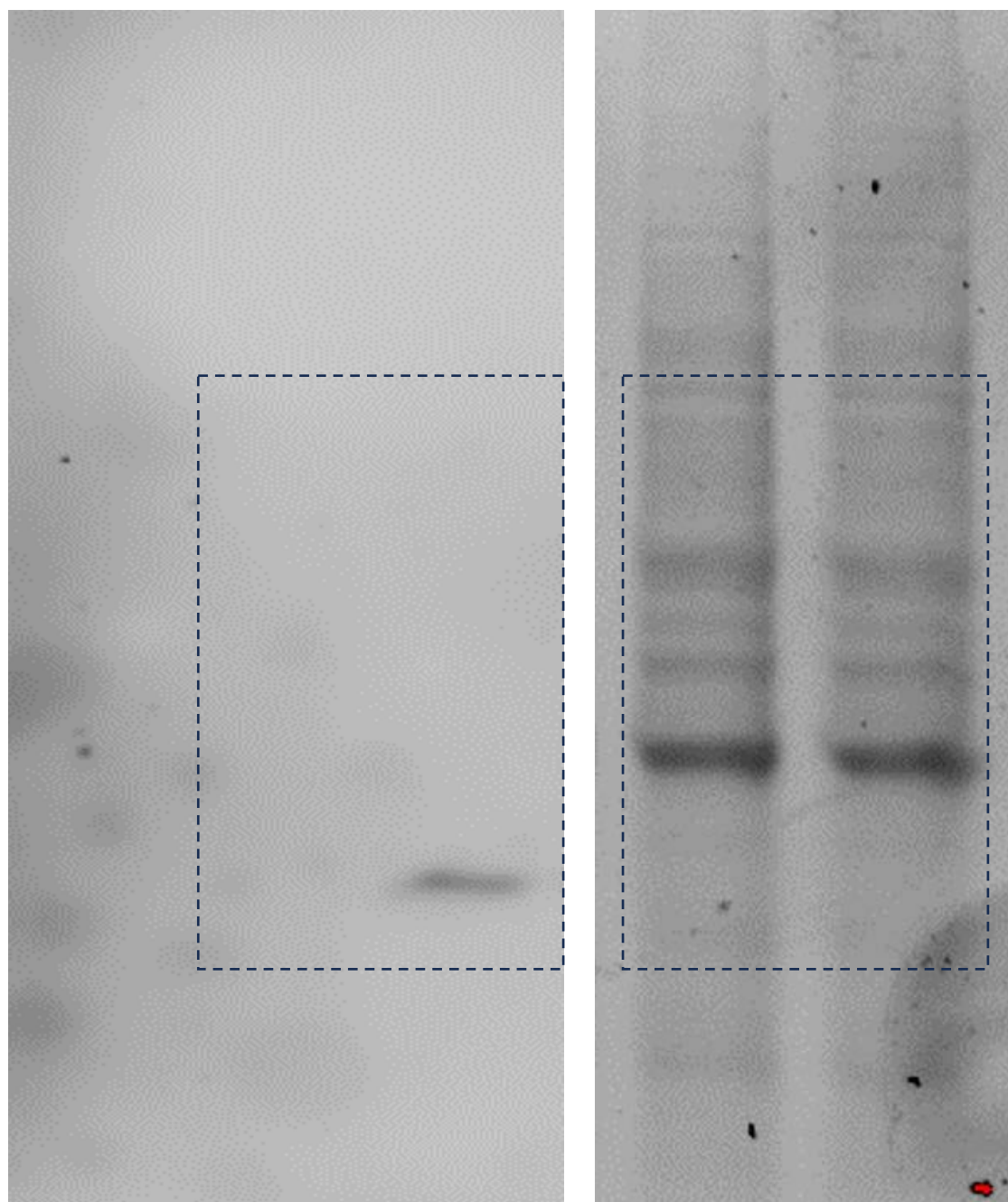

Fig. 4 D

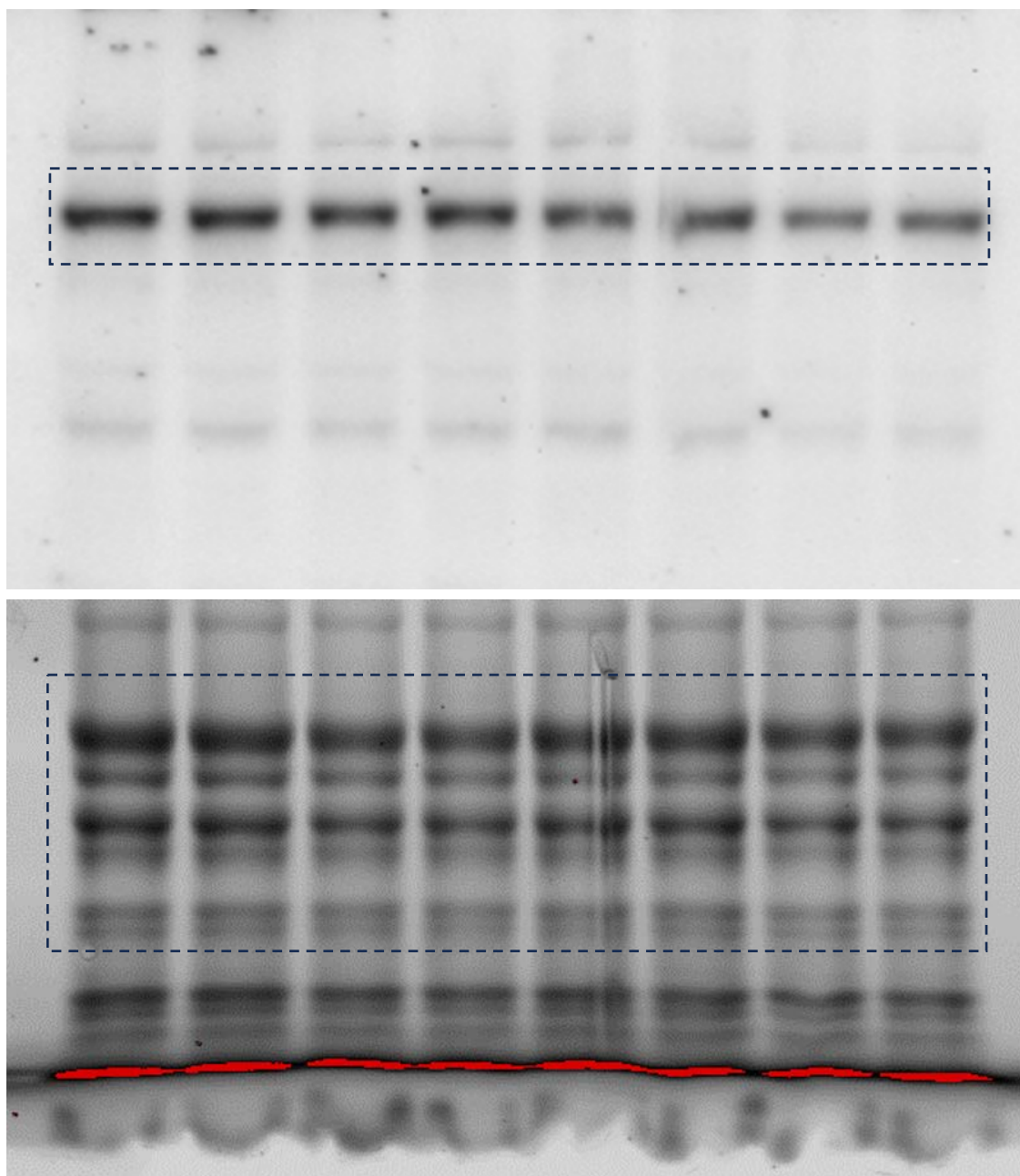

Fig. 6 A

Supplement: Supplementary file 1 — Additional file 1. Images of full-length blots/gels. [file 13287_2024_3669_MOESM1_ESM.pdf]
